# Effect of Framework Composition and NH_3_ on the Diffusion of Cu^+^ in Cu-CHA Catalysts Predicted by Machine-Learning Accelerated Molecular Dynamics

**DOI:** 10.1021/acscentsci.3c00870

**Published:** 2023-10-18

**Authors:** Reisel Millan, Estefanía Bello-Jurado, Manuel Moliner, Mercedes Boronat, Rafael Gomez-Bombarelli

**Affiliations:** † Department of Materials Science and Engineering, Massachusetts Institute of Technology, Cambridge, Massachusetts 02139, United States; ‡ Instituto de Tecnología Química, Universitat Politècnica de València-Consejo Superior de Investigaciones Científicas, Avenida de los Naranjos s/n, 46022 Valencia, Spain

## Abstract

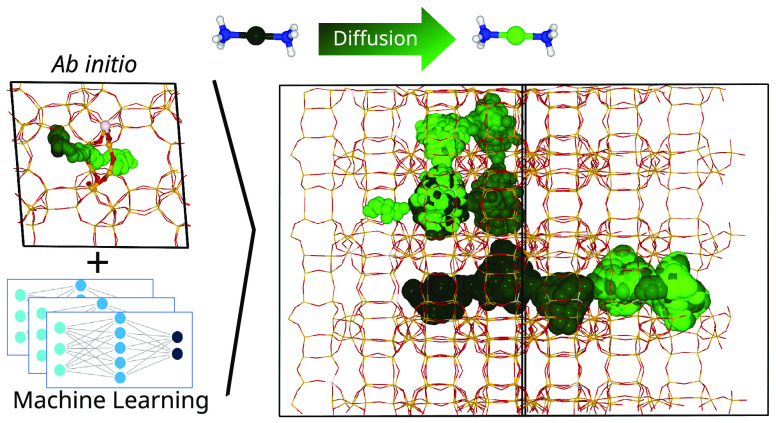

Cu-exchanged zeolites rely on mobile solvated Cu^+^ cations for their catalytic activity, but the role of the framework composition in transport is not fully understood. Ab initio molecular dynamics simulations can provide quantitative atomistic insight but are too computationally expensive to explore large length and time scales or diverse compositions. We report a machine-learning interatomic potential that accurately reproduces ab initio results and effectively generalizes to allow multinanosecond simulations of large supercells and diverse chemical compositions. Biased and unbiased simulations of [Cu(NH_3_)_2_]^+^ mobility show that aluminum pairing in eight-membered rings accelerates local hopping and demonstrate that increased NH_3_ concentration enhances long-range diffusion. The probability of finding two [Cu(NH_3_)_2_]^+^ complexes in the same cage, which is key for SCR-NOx reaction, increases with Cu content and Al content but does not correlate with the long-range mobility of Cu^+^. Supporting experimental evidence was obtained from reactivity tests of Cu-CHA catalysts with a controlled chemical composition.

## Abstract 2

Machine learning potentials reproduce accurately the ab initio dynamics of Cu^+^ cations in zeolite catalysts, allowing the exploration of previously unreachable time and length scales and chemical compositions.

## Introduction

Copper-exchanged zeolites play a crucial role as redox catalysts for some environmentally relevant processes, such as the partial methane oxidation to methanol or the selective catalytic reduction of nitrogen oxides with ammonia (NH_3_–SCR–NO_*x*_). In both cases, the small pore Cu-SSZ-13 zeolite with the CHA structure has been reported as an efficient catalyst.^1−11^

**Scheme 1:**
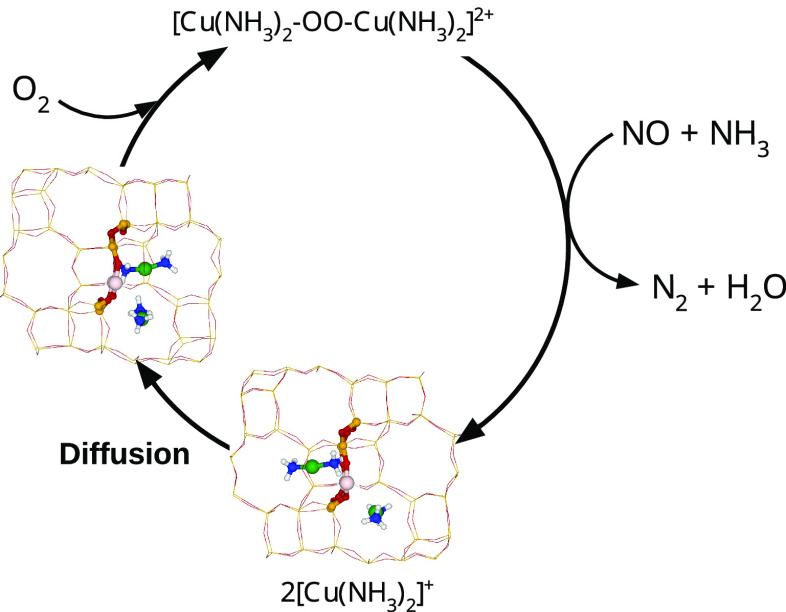
Illustration of the Low-Temperature SCR-NOx Redox Cycle

The NH_3_–SCR–NO_*x*_ reaction is currently employed for the removal of nitrogen oxides (NOx) from exhaust gases in diesel vehicles and stationary plants through a redox catalytic cycle in which Cu^+^ is oxidized to Cu^2+^ by O_2_, NO_2_, or NO + O_2_ and then reduced to Cu^+^ by the reaction of NH_3_ and NO forming harmless N_2_ + H_2_O (Scheme 1).^12−16^ This understanding of the reaction mechanism has enabled the development of optimized catalysts by tuning the framework topology, composition, and copper speciation. In the as-prepared catalysts, Cu^+^ and Cu^2+^ cations are directly coordinated to the zeolite framework forming heterogeneous active sites, while under reaction conditions NH_3_ solvates the Cu^+^ cations forming mobile [Cu(NH_3_)_2_]^+^ complexes that act as dynamic active sites, resembling homogeneous catalysts but within the confinement of the zeolite pores. At low temperature, that is, between 423 and 523 K, the oxidation step involves transient dimeric [Cu(NH_3_)_2_–OO–Cu(NH_3_)_2_]^2+^ species whose formation requires the simultaneous presence of two [Cu(NH_3_)_2_]^+^ monomers in the same *cha* cage. The hops between adjacent *cha* cages are modulated by size exclusion effects and also by the attractive interaction between the positively charged [Cu(NH_3_)_2_]^+^ complexes and the negatively charged framework Al sites.^8,9,15,17,18^ Thus, structural properties such as the Al content and distribution, Cu loading, and Brønsted acid site density as well as the interaction of the Cu active sites with the reactants, in particular, NH_3_, might affect the mobility of Cu cations and consequently the NH_3_–SCR–NO_*x*_ reaction rate. This has been evidenced by recent studies combining catalytic activity tests with *operando* XAS or EPR spectroscopy,^19−23^ and *ab initio* molecular dynamics (AIMD) simulations have been successfully applied to provide atomistic insight into the dynamic nature of the Cu^+^ cations under reaction conditions.^9,17,18^

The cost of AIMD simulations limits their applicability to a few selected systems at a time, at small length scales in the nanometer range and short time scales of ∼100 ps, while the faster classical force fields are not suited to describe the specific interactions involved in the systems investigated.^24^ For these reasons, the systematic exploration of parameters such as the Si/Al ratio, Al distribution, Cu/Al ratio, NH_3_ concentration, and the presence of Brønsted acid sites and compensating NH_4_^+^ cations has not yet been possible. Another possible avenue is to use machine learning potentials, which despite being slower than classical force fields allow access to the nanosecond scale.^25^

Machine learning (ML) has demonstrated broad applicability in materials science^26,27^ and heterogeneous catalysis.^28−31^ Machine learning potentials (MLPs), when trained with a sufficiently large and diverse data set, can match the accuracy of quantum chemistry methods at a fraction of the computational cost.^32−38^ This allows the study of larger and more realistic systems and more complex scientific problems,^27,39−41^ in particular, those requiring the use of molecular dynamics simulations.^42−44^ A broad variety of MLPs based on neural networks, so-called neural network potentials (NNPs), have been developed in the last few years (ANI,^45−48^ deep tensor neural networks,^49^ SchNet,^50^ DeepPotentialNet,^51^ MEGNet,^52^ DimeNet,^53^ OrbNet,^54^ PaiNN,^55^ NequIP^56^) and have been successfully used to study solid systems,^40,57−59^ ion diffusion,^60^ and chemical reactions,^61−63^ but the number of applications in the field of zeolite catalysis is still rather limited.^58,64,65^

Here, we leveraged these innovations and trained a NNP capable of describing [Cu(NH_3_)_2_]^+^ species in aluminosilicate CHA with varying composition and NH_3_ concentration. The trained NNP proved accurate and transferable, and acquiring all of the training data was less costly than one traditional AIMD simulation. Biased MD simulations reproduced free-energy profiles from DFT and provided insight into transport for over a dozen combinations of the Al distribution and the presence of NH_4_^+^. Unbiased MD simulations were scaled to thousands of atoms for nanoseconds and achieved a more realistic representation of the importance of Al density and distribution, Cu loading, and adsorbed NH_3_ in the mobility of Cu^+^ cations in Cu-CHA catalysts.

These results show that the activation free energy for [Cu(NH_3_)_2_]^+^ hops between adjacent cages is lower for windows containing Al pairs but also that this is a local effect with only a weak influence on long-range mobility. [Cu(NH_3_)_2_]^+^ migration to remote cages requires the simultaneous displacement of charge-compensating NH_4_^+^, which shows a lower mobility that is enhanced by excess NH_3_. Finally, simulations with large supercells show that the probability of finding two [Cu(NH_3_)_2_]^+^ complexes in the same cage, a prerequisite for the SCR-NOx reaction, increases with Cu loading and also with the Al content in the zeolite. We confirm these trends experimentally through catalytic tests of Cu-CHA samples with controlled Si/Al and Cu/Al ratios.

## Results and Discussion

### Neural Network Potential

**Figure 1:**
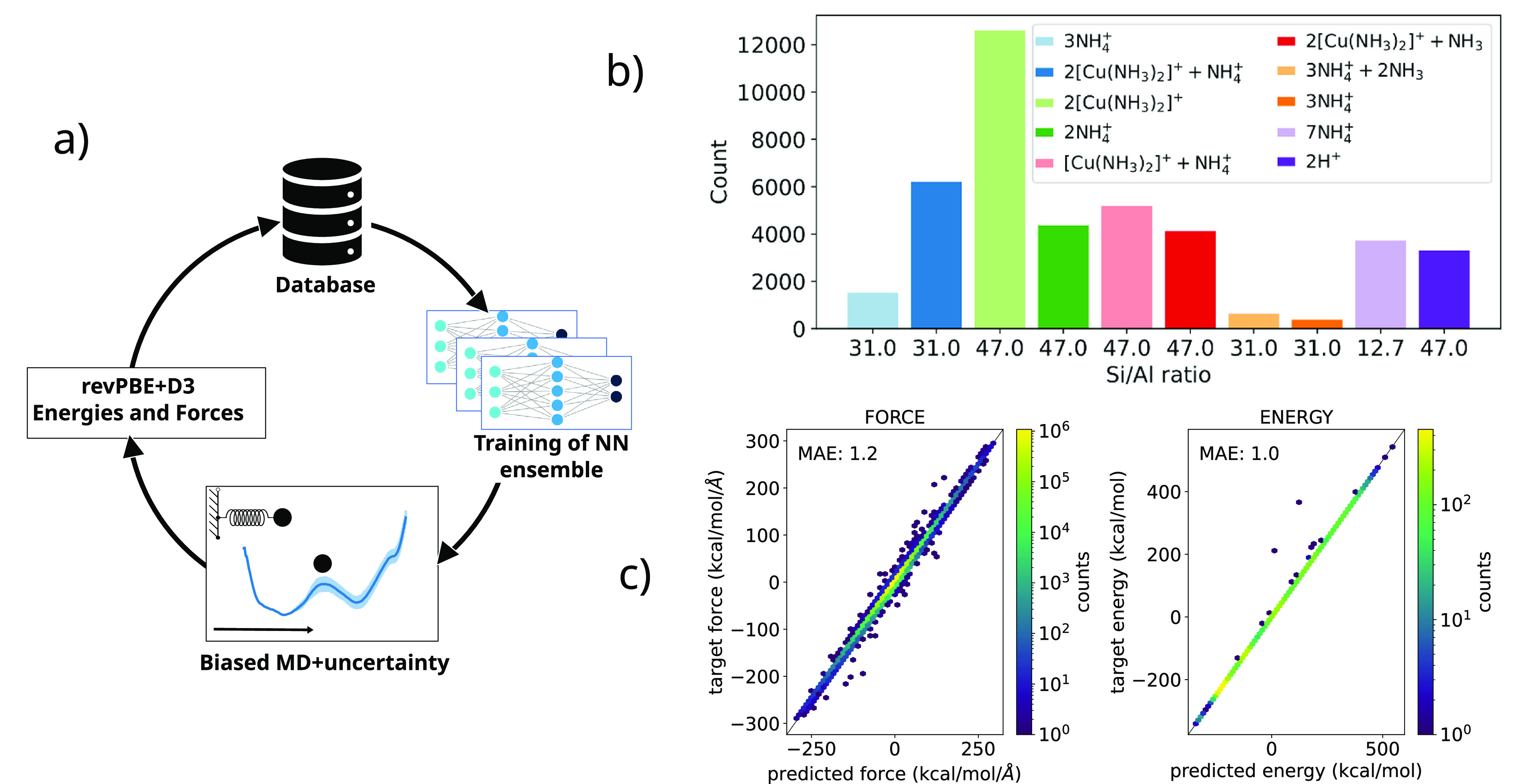
Neural network potential. (a) Illustration of the active learning cycle. At each iteration, an ensemble of NNPs is trained on the available labeled data contained in the database, initially obtained from previous PBE+D3 MD simulations. Then, biased MD trajectories are generated with these NNPs, and based on the force uncertainty of an ensemble of three NNPs, new geometries are collected, and their corresponding PBE+D3 energies and forces are computed and included in the data set. In the next loops, the newly generated data are combined with the existing data to train an updated NNP. (b) Distribution of the chemical composition of the 42K models included in the final data set, comprising models with Si/Al ratios of 13, 31 and 47, and with the negative charges arising from Al substitution compensated by [Cu(NH_3_)_2_]^+^, NH_4_^+^, or H^+^. (c) Correlation between the predicted and target energies (left) and forces (right) of the last-generation NNP.

NNPs are highly accurate, but they struggle to extrapolate outside their training data. In order to ensure robust and accurate production simulations, our NNP was trained on data gathered through multiple generations of active learning (AL) using a query-by-committee approach.^66−74^ A committee (ensemble) of NNPs was trained on the available labeled data at each iteration, and new data was collected based on the disagreement (variance) of the prediction of the committee members on newly generated geometries, as illustrated in Figure 1a. (See a more detailed description in the Methods section in the Supporting Information.) The first generation of the potential was trained on a randomly collected subset of the DFT data from a previous study^18^ and from three biased simulations performed with DFT at 423 K, used as reference ground truth. In total, there were ∼9000 geometries in the initial data set. This pretrained potential was then retrained in four active learning loops using the 2 × 2 × 2 triclinic supercell described in the Methods section and depicted in Figure S1. For each loop, biased MD trajectories were generated with the learned interatomic potential of the previous loop at temperatures of 298, 423, 500, and 550 K. The selection of the new geometries from the MD trajectories was carried out using as a criterion the force uncertainty from an ensemble of three NNPs. The variances among the forces within the ensemble of potentials were ranked in descending order, and the first geometries were selected to increase the data set in 10%. The nonphysical geometries and those with low uncertainty, <2 kcal/mol, were discarded. Up to this point, the data set contained only structures with the H_12_Al_2_Cu_2_N_4_O_192_Si_94_ composition (see Table 1 and the light-green bar in Figure 1b), where the negative charges generated by framework Al atoms were always compensated with [Cu(NH_3_)_2_]^+^ species so that the trained potential did not properly describe local environments of Al compensated with NH_4_^+^ or H^+^. The acquisition of new geometries with new compositions including NH_4_^+^ and H^+^ was performed using an adversarial attack^75^ for six more generations with NNPs trained on the last generation of active learning. Systems with only two Al substitutions per unit cell (Si/Al = 47) were included in the data set to control the distribution of Al pairs in the 8MR windows and to provide specific environments for regions with low local Al concentration. Then, five more generations of active learning were used, with biased MD simulations at temperatures ranging from 600 to 1000 K to force larger deviations from the equilibrium structures, thus ensuring a better configurational sampling. The last generation of the NNP was trained on a complete data set containing 42K revPBE+D3 force calculations on structural models containing from 290 to 323 atoms per supercell, with a diverse set of atomic local environments in which the negative charges arising from Al substitution were compensated with [Cu(NH_3_)_2_]^+^, NH_4_^+^, or H^+^^75−77^ as summarized in Table 1 and plotted in Figure 1b.

**Table 1:** Chemical Composition, Cationic Species, and Molecules Included in the Triclinic T_96_O_192_ Supercell Models Used for Active Learning and Adversarial Attack

**Formulas**	**Si/Al**	**Al**	**Si**	**[Cu(NH**_**3**_**)**_**2**_**]**^**+**^	**NH**_**4**_^**+**^	**NH**_**3**_	**H**^**+**^
H_2_Al_2_O_192_Si_94_	47	2	94	0	0	0	2
H_8_Al_2_N_2_O_192_Si_94_	47	2	94	0	2	0	0
H_12_Al_3_N_3_O_192_Si_93_	31	3	93	0	3	0	0
H_15_Al_3_N_4_O_192_Si_93_	31	3	93	0	3	1	0
H_18_Al_3_N_5_O_192_Si_93_	31	3	93	0	3	2	0
H_28_Al_7_N_7_O_192_Si_89_	12.7	7	89	0	7	0	0
H_12_Al_2_Cu_2_N_4_O_192_Si_94_	47	2	94	2	0	0	0
H_10_Al_2_Cu_1_N_3_O_192_Si_94_	47	2	94	1	1	0	0
H_18_Al_2_Cu_2_N_6_O_192_Si_94_	47	2	94	2	0	2	0
H_16_Al_3_Cu_2_N_5_O_192_Si_93_	31	3	93	2	1	0	0

The active learning strategy was capable of automatically adding new, diverse, and informative chemical environments to the training pool at each of the preselected compositions through a combination of MD and uncertainty quantification. It generated informative training data for a number of chemical processes that occur during the reaction but were not present in the initial training data. These include adsorption and protonation of NH_3_ on the Brønsted acid sites to form NH_4_^+^ cations, exchange between a gas-phase NH_3_ molecule and one of the two NH_3_ ligands of the [Cu(NH_3_)_2_]^+^ complex, and proton transfer from NH_4_^+^ to NH_3_. The diffusion of [Cu(NH_3_)_2_]^+^ complexes through the 8R windows that connect adjacent *cha* cages has a higher activation barrier. Therefore, representative training data was obtained through the same enhanced sampling approach as the production simulations (Figure S2).

This strategic combination of biased MD with uncertainty quantification allowed efficient sampling of the relevant regions on the PES with a small and diverse number of DFT evaluations. Figure S3 illustrates the structural diversity in the final data set by means of a 2D projection of the local chemical environments around each Al atom in our data using UMAP^78^ on the feature vectors learned by the NNP.^79^ Atoms with similar local environments have similar feature vectors and appear close to the UMAP plot. The overlap among the chemical compositions suggests a nearly continuous sampling of the Al local environment.

Figure 1d shows the correlation between predicted and target energies and forces for a held-out test set. The mean absolute error of the predicted energies and forces are 0.98 and 1.2 kcal/mol/Å, respectively, indicating that the NNP is capable of predicting the energies and forces with chemical accuracy.

### Effect of Al Distribution on [Cu(NH_3_)_2_]^+^ Diffusion through 8R Windows from Biased Simulations

**Figure 2:**
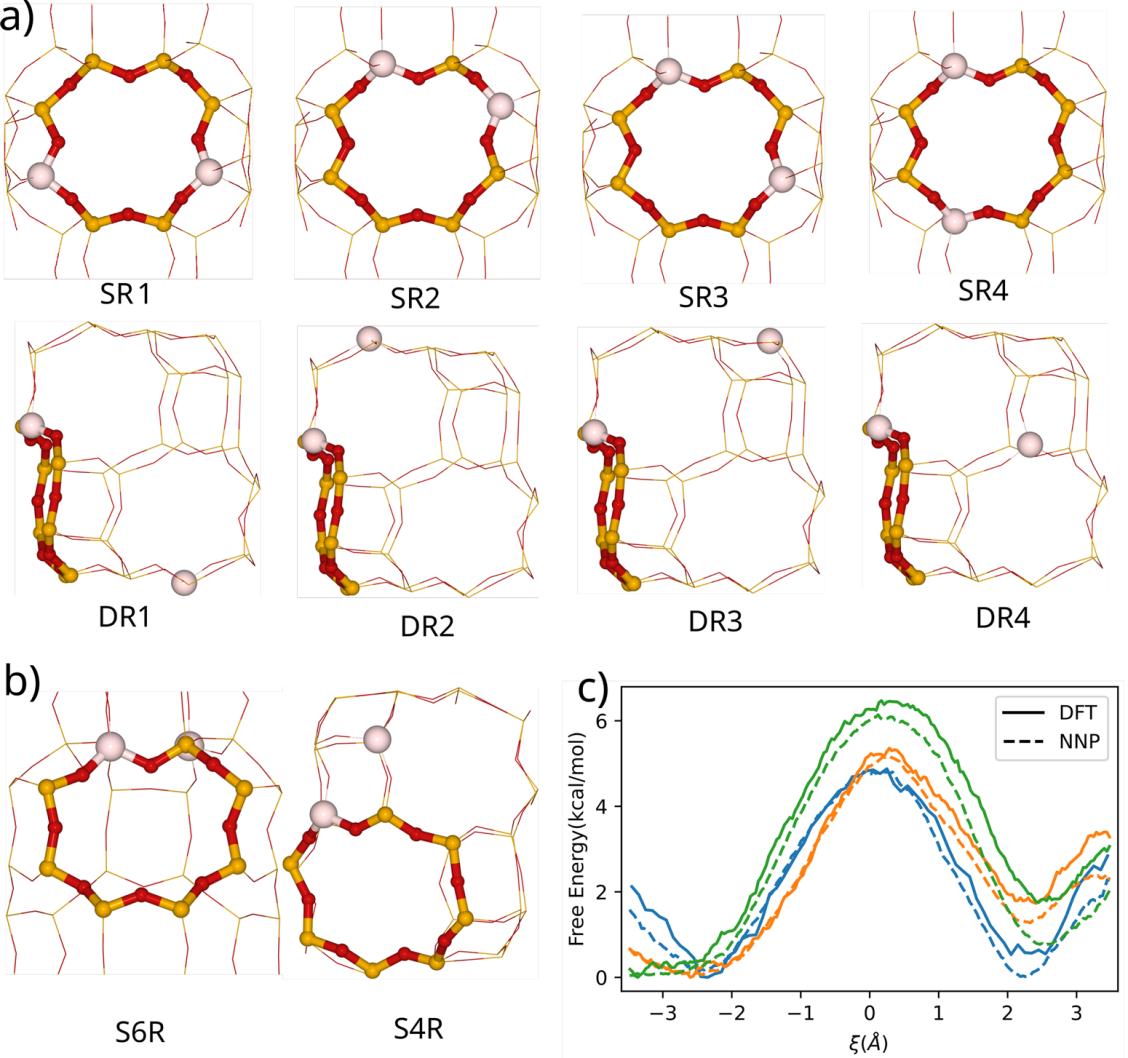
Structural models and accuracy of NNP biased simulations. (a, b) Representation of the different Al pair distributions used in the biased simulations. Si and O atoms are depicted as orange and red sticks, respectively. Al atoms are depicted as light-brown balls. The Si and O in the 8R window through which the [Cu(NH_3_)_2_]^+^] complex diffuses are highlighted as orange and red balls. (c) Comparison of free-energy profiles for [Cu(NH_3_)_2_]^+^ diffusion through the 8R window of SR1 (orange), SR4 (blue), and DR2 (green) systems obtained from DFT (solid line)- and NNP (dashed line)-based biased simulations at 423 K.

The favorable speed of the NNP accelerates US MD simulations by orders of magnitude over DFT, and it enabled a systematic exploration of the role of Al distribution in well-converged simulations. Ten different structural models with the same H_12_Al_2_Cu_2_N_4_O_192_Si_94_ composition corresponding to Si/Al = 47 but with different Al distributions were built (Figure 2a,b). The two Al atoms were placed either in the same 8R window (structures labeled SR1, SR2, SR3, and SR4), in different 8R windows (structures labeled DR1, DR2, DR3, and DR4) in the same 4R (S4R), or in the same 6R (S6R), and each framework Al was compensated with a [Cu(NH_3_)_2_]^+^ cationic complex (entry 7 in Table 1).

Because the error of the predicted forces is not a sufficient metric for true performance in production simulations,^25^ the NNPs were further validated by comparing NNP and DFT free-energy profiles for [Cu(NH_3_)_2_]^+^ diffusion through the 8R window of SR1, SR4, and DR2 models. Due to the high computational cost of producing reference DFT biased simulations, a smaller hexagonal 126-atom unit cell was used. The free-energy profiles for SR1, SR4, and DR2 (Figure 2c) and the activation free energies (*ΔF*_act_) calculated as the energy difference between the maximum and the minimum on the profile, 5.2 and 5.3 kcal/mol for SR1, 4.8, and 4.8 kcal/mol for SR4, and 6.1 and 6.4 kcal/mol for DR2, are in excellent agreement at both computational levels.

The smoother NNP traces are a consequence of more abundant sampling (4.8 ns total as compared to 0.8 for DFT) given the advantageous computational cost of NNPs (20 ps of AIMD required over a week on CPU Intel(R) Xeon(R) E5-2650 cores as compared to 20 min on a Tesla V100-32 GB GPU for NNPs).

**Figure 3:**
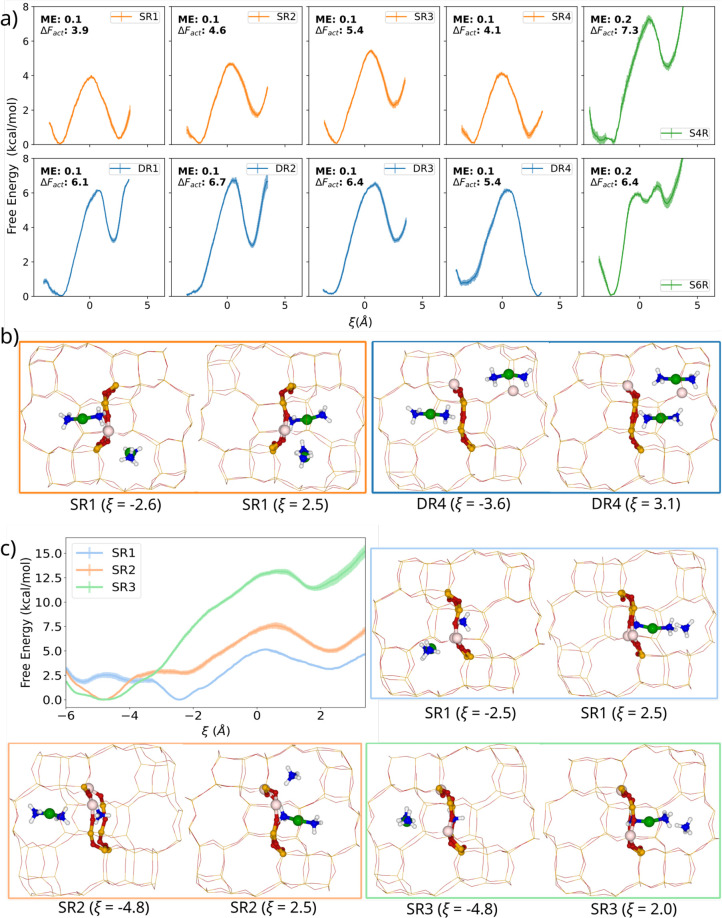
NNP biased simulations of [Cu(NH_3_)_2_]^+^ diffusion. (a) Free-energy profiles for the diffusion of one [Cu(NH_3_)_2_]^+^ complex from cage A to neighboring cage B occupied by another [Cu(NH_3_)_2_]^+^ complex. (b) Snapshots of the initial and final minimum states corresponding to the diffusion of [Cu(NH_3_)_2_]^+^ through the 8R windows of SR1 and DR4 systems at 423 K. (c) Free-energy profiles for [Cu(NH_3_)_2_]^+^ diffusion between neighboring cages in the presence of one NH_4_^+^ cation initially placed in the 8R to be crossed in three systems with different Al distributions obtained from NNP-based biased simulations at 423 K and snapshots of the initial and final minimum states in the systems SR1, SR2, and SR3. Si, O, Al, H, Cu, and N atoms are depicted in orange, red, light brown, white, green, and blue, respectively. The atoms in the 8R window through which [Cu(NH_3_)_2_]^+^ diffuses are highlighted.

Then, the free-energy profiles for [Cu(NH_3_)_2_]^+^ diffusion between neighboring cages in the 10 systems depicted in Figure 2a,b were obtained from NNP biased simulations at 423 K using the larger H_12_Al_2_Cu_2_N_4_O_192_Si_94_ models. The profiles are plotted in Figure 3, and the corresponding values of activation (*ΔF*_act_) and reaction (*ΔF*) free energies are summarized in Table S1. The shaded area in each profile shows the standard error calculated from three independent simulations using three different NNPs trained on the same data set. The average value of the standard error, 0.1 kcal/mol in all cases, indicates a low uncertainty in the prediction of the free energy and well-converged statistics.

For the systems with two Al atoms in the same 8R (orange profiles in Figure 3a), *ΔF*_act_ values range from 3.9 to 5.4 kcal/mol and the reaction is slightly endergonic with *ΔF* values between 0.4 and 2.3 kcal/mol. In all other cases, *ΔF*_act_ is higher than 6 kcal/mol and *ΔF* is larger than 3 kcal/mol, with the only exception of the DR4 system for which the process is slightly exergonic. The Al distribution in the DR4 model is the same as in the SR1 model, but the diffusion of the [Cu(NH_3_)_2_]^+^ complex proceeds through different 8R windows of the same model, highlighted in Figure 3b. In both cases, the stability of the final state with the two [Cu(NH_3_)_2_]^+^ complexes in the same cage is similar to that of the initial state with the two complexes in different cages, which suggests that this particular Al distribution might favor the formation of the [Cu(NH_3_)_2_]^+^–OO–[Cu(NH_3_)_2_]^+^ dimers involved in the low-temperature NH_3_–SCR–NOx reaction. In contrast, distributions with two Al atoms in the same 4R or 6R hinder the formation of such dimeric intermediates because only one of the two [Cu(NH_3_)_2_]^+^ complexes can stay near the Al atoms, while the second [Cu(NH_3_)_2_]^+^ is forced to remain too close to the first, resulting in its diffusion through a different 8R to another empty cage. These results demonstrate that the Al distribution affects the movement of [Cu(NH_3_)_2_]^+^ species between cages and the stability of pairs of [Cu(NH_3_)_2_]^+^ complexes in the same cage and points to a positive effect of Al pairs in 8R on the rate of the low-temperature NH_3_–SCR–NOx reaction.

### Effect of NH_4_^+^ on the Diffusion of [Cu(NH_3_)_2_]^+^ through 8R Windows from Biased Simulations

The mobility of [Cu(NH_3_)_2_]^+^ complexes within the zeolite microporous structure is affected by the presence of other molecules involved in the reaction, among which NH_3_ is the most abundant and the one with the largest impact on diffusion and reactivity.^17,18,21,22,80,81^ Under reaction conditions, NH_3_ is readily protonated on the Brønsted acid sites forming NH_4_^+^ cations that remain coordinated to the framework AlO_4_^–^ units and might partly block the diffusion of [Cu(NH_3_)_2_]^+^ complexes through the 8R windows. To analyze this possibility, we first performed NNP biased simulations at 423 K using the previously described H_10_Al_2_Cu_1_N_3_O_192_Si_94_ models with two framework Al atoms compensated now with one [Cu(NH_3_)_2_]^+^ complex initially placed in the center of cavity A (see Figure S2) and one NH_4_^+^ cation initially placed in the plane of the 8R through which [Cu(NH_3_)_2_]^+^ will diffuse. The free-energy profiles obtained for the three Al distributions considered (SR1, SR2, and SR3), as plotted in Figure 3c, are clearly different from those depicted in Figure 3a, and the calculated activation free energies for [Cu(NH_3_)_2_]^+^ diffusion are also reflective of how the strong coordination of NH_4_^+^ modifies transport.

In the previous simulations without NH_4_^+^ the most stable minimum for the initial state occurs at ξ ≈ −2.5 Å, with [Cu(NH_3_)_2_]^+^ relatively close to the 8R, which is the case only for SR1 in the presence of NH_4_^+^ (blue profile in Figure 3c). In SR2 and SR3 models (orange and green profiles), the most stable minimum lies at ξ ≈ −4.8 Å, with the [Cu(NH_3_)_2_]^+^ complex closer to the center of the cavity and at a larger distance from the 8R to be crossed. The calculated *ΔF*_act_, 5.2, 7.6, and 13.1 kcal/mol for SR1, SR2, and SR3, respectively, and *ΔF* values, 3.2, 5.0, and 11.4 kcal/mol for SR1, SR2, and SR3, respectively, are higher than those obtained for the corresponding systems in the absence of NH_4_^+^. The snapshots of the final state at ξ ≈ 2.5 Å for SR1 and SR2 in Figure 3c show that the NH_4_^+^ cation has been displaced from its initial position in the plane of the 8R to a position relatively close to one of the AlO_4_^–^ units. In SR3, however, the NH_4_^+^ cation has been displaced to the opposite side of the cage, far from the two AlO_4_^–^ sites present in the model, which would explain the instability of the system. The deviation across replicate profiles is wider in some regions, which we attribute to the fact that our simulations occasionally, but not exhaustively, sample the spontaneous reversible deprotonation of NH_4_^+^ cations to form NH_3_ and a Brønsted acid site, which is typically not accessible to traditional simulations.

Altogether, the results from the biased simulations suggest a potential blocking effect of the NH_4_^+^ cations. However, their own mobility, as either NH_4_^+^ or NH_3_ following proton transfer to deprotonated AlO_4_^–^, and possible migration from the 8R toward other nearby AlO_4_^–^ units that are not present in this model might modify this conclusion. The NNPs developed here open the possibility of running long-time unbiased MD simulations on larger systems with more realistic chemical compositions, allowing one to capture the dynamics of [Cu(NH_3_)_2_]^+^ and NH_4_^+^ globally and to observe long-range diffusion of both cationic species.

### Long-Range Diffusion of NH_4_^+^ and [Cu(NH_3_)_2_]^+^ Species from Unbiased MD Simulations

A large T_768_O_1536_ supercell with a dimension of ∼37 Å was used to construct eight models representing three Al contents, low (L, Si/Al ≈ 30) with 26 Al atoms in the unit cell, medium (M, Si/Al ≈ 14) with 50 Al atoms in the unit cell, and high (H, Si/Al ≈ 10) with 68 Al atoms in the unit cell, as described in detail in the Methods section and Figure S4 in the Supporting Information. The framework Al were compensated with combinations of [Cu(NH_3_)_2_]^+^, NH_4_^+^, and H^+^ as summarized in Table S2. Each model’s name contains a letter indicating the Al content (L, M, or H) followed by two numerical values indicating the number of [Cu(NH_3_)_2_]^+^ and NH_4_^+^ compensating cations. Thus, L(20-6) indicates low Al content, with 26 Al atoms in the unit cell compensated with 20[Cu(NH_3_)_2_]^+^ and 6NH_4_^+^ cations. Unbiased MD simulations were conducted for at least 5 ns on each ∼2000-atom system using a slightly higher *T*, 500 K, in order to enhance the mobility of the [Cu(NH_3_)_2_]^+^ cations and increase the probability of hopping events between neighboring cavities.

**Figure 4:**
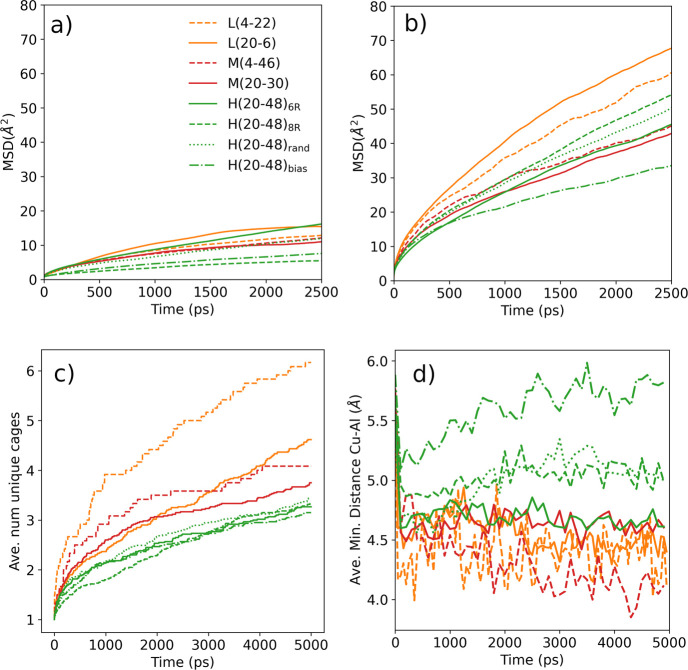
Mobility of NH_4_^+^ and [Cu(NH_3_)_2_]^+^. (a) Mean square displacement (MSD) of the N atoms in NH_4_^+^ and (b) MSD of the Cu atoms in the [Cu(NH_3_)_2_]^+^ complexes obtained from NPP-based unbiased MD simulations at 500 K. (c) Number of distinct cages visited by the Cu atoms. The apparent contradiction with the MSD plots is explained by the fact that the diffusion of [Cu(NH_3_)_2_]^+^ does not occur along a preferential direction. One Al pair in an 8R can accelerate the diffusion of [Cu(NH_3_)_2_]^+^ between two neighboring cages in multiple forward and backward steps so that only two distinct cages are visited by this complex. (d) Average minimum Cu–Al distances. All plots were obtained from the same simulations. See the Supporting Information for details on the calculation of MSD.

Figure 4a,b tracks the time evolution of the mean square displacements (MSDs) of the N atoms in the NH_4_^+^ cations and of the Cu atoms in the [Cu(NH_3_)_2_]^+^ complexes, respectively. While both species have a net charge of +1, [Cu(NH_3_)_2_]^+^ is much more mobile while NH_4_^+^ cations are more closely attached to the AlO_4_^–^ units (Figures S5 and S6). This is because the positive charge in the [Cu(NH_3_)_2_]^+^ complexes is highly shielded by the two NH_3_ ligands.

The similarity among the MSD profiles of N atoms suggests that the mobility of NH_4_^+^ cations is rather independent of the zeolite framework composition and Cu content (Figure 4a), while the MSD traces for Cu (Figure 4b) suggest slightly lower mobility of [Cu(NH_3_)_2_]^+^ in the systems with high Al content (Si/Al ≈ 10, Figure 4b). The trends are similar in the number of distinct cages visited by the [Cu(NH_3_)_2_]^+^ complex over time (Figure 4c), but they are more stratified and more clearly show an increase in mobility with decreasing copper content (orange and red dashed lines higher than solid lines in Figure 4c). In 5 ns, each [Cu(NH_3_)_2_]^+^ complex visits on average fewer than three different cages in the models with high Al content, which increases to three to four for intermediate Al and reaches a maximum of over five different cages visited for the L4 model, which has the lowest Al content and thus few Al pairs in 8R. This is in apparent contradiction to the biased simulation results for small models, which showed a lower hopping free-energy barrier for Al pairing in 8R. A potential explanation is that the hopping landscape is statically and dynamically heterogeneous, with the local chemical environment of each initial and final cage and transient cage occupation by other mobile molecules influencing the mobility of copper complexes. Figure S5 shows the diversity in length and tortuosity in example trajectories of Cu atoms inside the zeolite microporous structure. While MSD profiles representing the average movement of the [Cu(NH_3_)_2_]^+^ species are relatively similar across catalyst models (∼40–60 Å^2^), the local mobility of each individual [Cu(NH_3_)_2_]^+^ complex depends on its local chemical environment.

Once the influence of Al content was established, we analyzed the effect of NH_4_^+^ on the mobility of [Cu(NH_3_)_2_]^+^ complexes in systems with a constant Si/Al ratio. Increasing the amount of NH_4_^+^ from 30 to 46 cations (compare M(20–30) with M(4–46) models in Figure 4c) or from 6 to 22 cations (compare L(20–6) with L(4–22) in Figure 4c) increases the number of cages visited, suggesting a positive effect of NH_4_^+^ on the long-range diffusion of [Cu(NH_3_)_2_]^+^. A proposed explanation is that adsorbed NH_4_^+^ shields the attractive interaction between the AlO_4_^–^ anionic sites and the [Cu(NH_3_)_2_]^+^ complexes. Each NH_4_^+^ forms two strong hydrogen bonds with the AlO_4_^–^ site, hence the lower mobility of NH_4_^+^, while [Cu(NH_3_)_2_]^+^ interacts with the zeolite through the H atoms of the coordinating NH_3_ molecules. This “crowding” of the anionic sites by the harder NH_4_^+^ can be observed statistically in the simulations. The average distances between the Cu atoms and the closest framework Al atoms plotted in Figure 4d are ∼4.5 Å in the L and M models (orange and red lines) and increase to ∼5.0 Å in the systems with higher Al content and thus a larger amount of charge-balancing NH_4_^+^ (green lines). The H(20–48)_bias_ model has a heterogeneous Al distribution, and its anomalously high Cu–Al distance is due to [Cu(NH_3_)_2_]^+^ complexes in Al-poor regions.

Previous studies have suggested that [Cu(NH_3_)_2_]^+^ migration is fast only among the three cages sharing a common framework Al, while long-range diffusion to nonadjacent cages is limited to ∼9 Å due to the decaying electrostatic interaction between the [Cu(NH_3_)_2_]^+^ and the anionic AlO_4_^–^ site.^9,18,20^ This argument is rigorously true when the final cages contain no additional Al sites, as in our model systems from the first section. In real systems, however, the long-range diffusion is easily explained through a sequence of local steps combining the crossing of 8R windows into adjacent Al-containing cages, followed by the exchange of NH_4_^+^ as a compensating cation (Scheme S1 in the Supporting Information). The low mobility of NH_4_^+^ revealed by the present simulations suggests that a limiting factor for such long-range diffusion of [Cu(NH_3_)_2_]^+^ complexes is the slower rate of countermigration of the charge-compensating NH_4_^+^. While [Cu(NH_3_)_2_]^+^ could act as a migrating compensating cation, this results in no net migration of Cu.

To explore this hypothesis, two additional MD simulations of 5 ns were run using two modified M20 models, one of them containing 30 protons as compensating cations, labeled as M20-H+, and another one with 60 additional NH_3_ molecules added to the system, labeled as M20-NH3. Protons on the Brønsted acid sites are fairly static and localized within the four oxygen atoms directly attached to Al, so the long-range charge-compensation diffusion of H^+^ should be hindered. In contrast, additional NH_3_ should facilitate the movement of the positive charges via proton transfer from NH_4_^+^ to NH_3_ via a Grotthuss-like chain of proton transfers, thus allowing faster charge compensation between separated AlO_4_^–^ units without the need to physically displace the strongly attached NH_4_^+^ cations.

**Figure 5:**
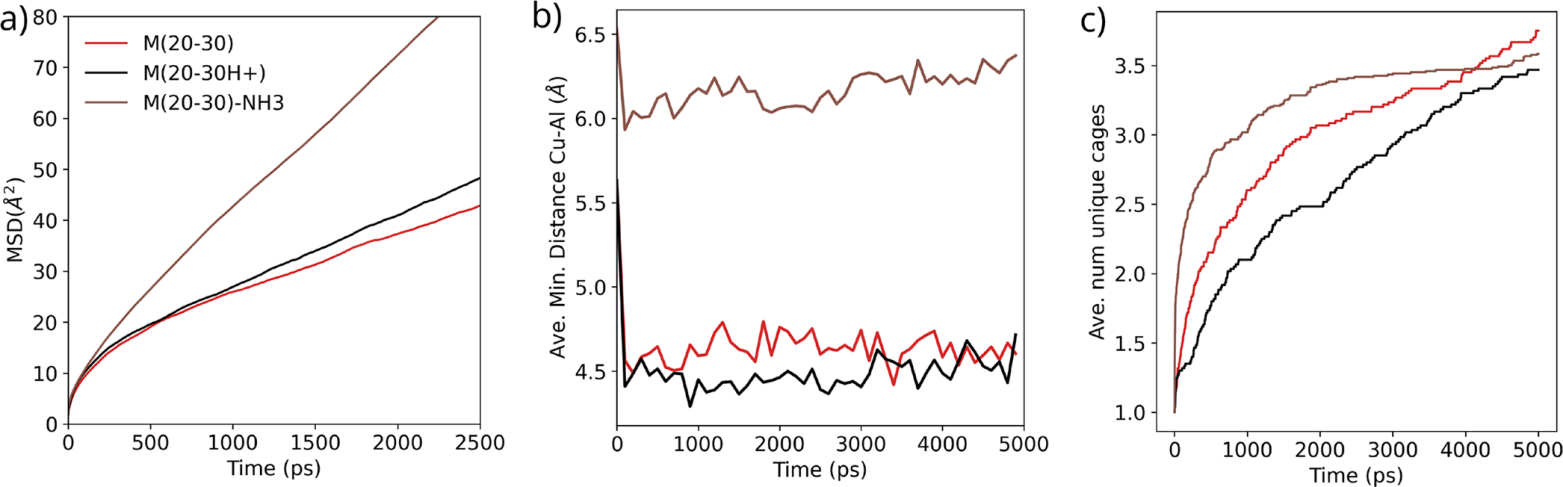
Influence of framework charge shielding on the mobility of [Cu(NH_3_)_2_]^+^. (a) Mean square displacement (MSD) of Cu atoms in the [Cu(NH_3_)_2_]^+^ complexes, (b) average minimum Cu–Al distances, and (c) number of distinct cages visited obtained from NPP-based unbiased MD simulations at 500 K in models with different charge-compensating cations and additional NH_3_ molecules. See the Supporting Information for details on the calculation of MSD.

The plots in Figure 5 confirm this hypothesis. The MSD of Cu atoms in Figure 5a does not change when NH_4_^+^ cations are substituted by protons (black lines) with a similar electrostatic shielding effect as NH_4_^+^. However, the Cu mobility increases significantly in the presence of excess NH_3_ molecules (brown lines), suggesting that free NH_3_ facilitates the charge re-equilibrium following [Cu(NH_3_)_2_]^+^ crossing 8R. The average distances between the Cu atoms and the closest framework Al atoms increase from ∼4.5 to ∼6.0 Å with the added NH_3_ (Figure 5b). Finally, the number of distinct cages visited by [Cu(NH_3_)_2_]^+^ (Figure 5c) indicates again a slightly lower long-range mobility of [Cu(NH_3_)_2_]^+^ in the model containing Brønsted acid sites and enhanced diffusion in the presence of an excess of NH_3_.

Our results provide theoretical backing to recent experimental observations of the enhancing effect of gas-phase NH_3_ during the solid-state ion exchange of copper using mixtures of copper oxides and zeolites, which allows the fast preparation of Cu-exchanged zeolites at low temperatures (473–523 K).^80,81^ After ∼4000 ps, the three models reached similar steady states with ∼3.5 distinct cages being visited by each complex, corresponding to the final state of the ion-exchange process with a random distribution of [Cu(NH_3_)_2_]^+^ complexes occupying the whole unit cell.

### Bimolecular Complexes and Mechanistic Implications for the NH_3_–SCR–NOx Reaction

**Figure 6:**
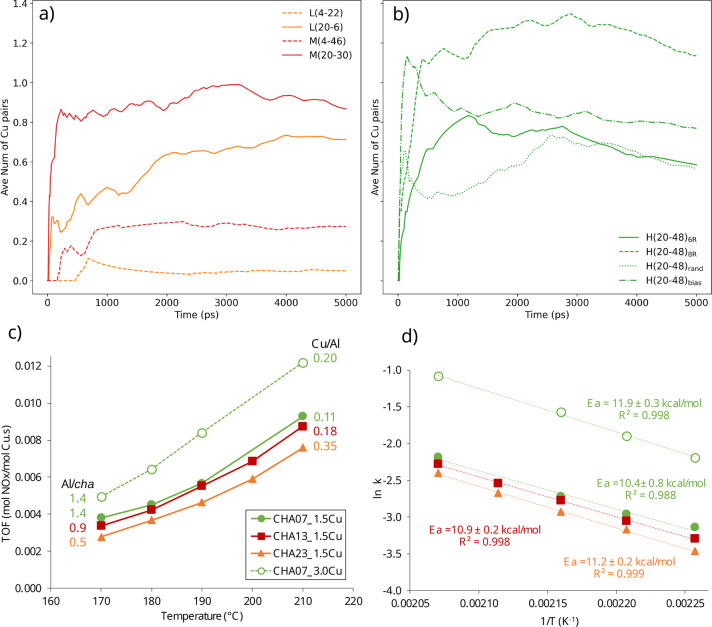
Probability of formation of the active sites and catalytic results for the NH_3_-SCR-NOx reaction. (a,b) Time evolution of the average number of [Cu(NH_3_)_2_]^+^ pairs in the same cage for the eight systems in Figure S4. The geometric centers of every cage in the unit cell are determined, and two Cu atoms are considered to be in the same cage when both have the same cage center as the nearest cage center. (c) Turnover frequency (TOF) values on a per Cu ion basis for the ∼1.5 wt % Cu-containing zeolites with different Si/Al molar ratios and the CHA07 sample (Si/Al ≈ 7) with two different Cu contents (∼1.5 and ∼3 wt % Cu). (d) Arrhenius plots for the same experiments including the corresponding values of apparent activation energy obtained from the slope of each plot.

According to the proposed mechanism,^9^ the reaction rate depends directly on the number of dimeric intermediates formed by the pairing of two [Cu(NH_3_)_2_]^+^ complexes in the same cage. Figure 6a,b tracks the time evolution of the average number of [Cu(NH_3_)_2_]^+^ pairs, that is, two [Cu(NH_3_)_2_]^+^ ions simultaneously in the same cage, formed in each of the eight models analyzed in the previous section. As expected,^9,19,20^ the number of [Cu(NH_3_)_2_]^+^ pairs depends directly on the total amount of Cu in the system (compare yellow and red dashed lines with yellow and red full lines). More interestingly, for a given Cu content, the number of [Cu(NH_3_)_2_]^+^ pairs formed along the simulation is systematically larger in the systems with higher Al content, in agreement with recent work by Krishna et al. showing that the fraction of Cu^+^ cations that can be oxidized by O_2_ increases with increasing Al content in Cu-CHA zeolites of varying composition.^20^ On the other hand, the four models with the same Cu content, same Si/Al ratio of ∼10, and different Al distributions H(20–48)_6R_, H(20–48)_8R_, H(20–48)_rand_, and H(20–48)_bias_ (see Methods section, Figure S4, and Table S2) exhibit quite similar but not fully equivalent behavior. The time evolution plots in Figure 6b suggest a higher probability of [Cu(NH_3_)_2_]^+^ pairing in the H(20–48)_8R_ models containing two framework Al atoms in the same 8R, in agreement with the lower activation free energies (Δ*F*_act_) obtained for [Cu(NH_3_)_2_]^+^ diffusion through these Al-pair-containing 8R windows.

### Experimental Validation for the Low-T NH_3_-SCR-NOx Reaction Catalyzed by Cu-CHA Zeolites with Controlled Composition

To experimentally validate the computational predictions, three CHA samples with different Si/Al molar ratios ranging from 7.3 to 23.3, which translates to a broad range of 1.4 to 0.5 Al sites per *cha* (see Table S3 in the Supporting Information) were synthesized as described in the Methods section. Then, the same Cu loading (∼1.5 wt % Cu) was introduced within the three CHA materials, which resulted in a similar amount of initial Cu atoms per *cha* cage, ∼0.17, but different Cu/Al ratios (from 0.11 to 0.35, see Table S4). In addition, the CHA sample with a Si/Al ratio of ∼7.3 was also loaded with 3.0 wt % Cu, resulting in an additional sample with an increased number of initial Cu atoms per *cha* cage (0.3). The Si/Al ratios in these samples and in the industrial catalysts are lower than those considered in some systems of the training data set and in some simulations. The reason is that in macroscopic systems the Al atoms are not uniformly distributed along the crystal, and the training of the NNP should include local environments with higher and lower Si/Al ratios to avoid extrapolations.

The catalytic tests to evaluate the low-temperature SCR-NOx activity of the different Cu-CHA materials were performed at very high space velocities (1800000 mL/h·g of catalyst) to ensure low NO conversions (<20%). Turnover frequency values (TOF) were obtained for each Cu-CHA sample at five different temperatures (Figure 6c). The TOF values obtained for the three catalysts with different Si/Al molar ratios and the same Cu content (∼1.5 wt % Cu) exhibit a continuous activity enhancement as the Al/*cha* cage ratio increases from 0.5 to 1.4 (see Figure 6c), in agreement with the theoretical conclusion that the probability of simultaneously finding two [Cu(NH_3_)_2_]^+^ complexes in the same *cha* cage increases with increasing the Al content in the zeolite. A comparison of the TOF values obtained for samples with the same Si/Al ratio and different Cu content (full and dotted green lines in Figure 6c) or even with similar Cu/Al ratios and different Cu content (red and dotted green lines in Figure 6c) confirms that the catalytic activity clearly improves with increasing the Cu/*cha* ratio, in good agreement with the theoretical conclusion that the probability of forming [Cu(NH_3_)_2_]^+^ pairs in the same *cha* cage directly correlates with the total amount of Cu in the system.

The experimental apparent activation energies (Figure 6d) are similar for all samples irrespective of the Si/Al ratio, Cu content, or catalytic activity, ranging from 10.4 ± 0.8 to 11.9 ± 0.3 kcal/mol. The catalytic activity measured by the TOF and normalized by Cu content, however, increases in parallel with the calculated likelihood of two copper encounters in the same cage (Figure 6a,b). This supports the argument that the formation of [Cu(NH_3_)_2_]^+^ pairs in the same *cha* cage is responsible for the generation of the binuclear active sites that catalyze the reaction, although copper diffusion may not necessarily be the rate-determining step of the global process.

## Conclusions

Biased and unbiased MD simulations using a newly trained NNP have achieved high accuracy, chemical diversity, and good length and time scales, allowing the systematic investigation of the influence of catalyst composition and adsorbed NH_3_ on the mobility of [Cu(NH_3_)_2_]^+^ cations in Cu-CHA catalysts.

Biased simulations on small systems showed that single [Cu(NH_3_)_2_]^+^ cation hops between adjacent *cha* cages are very sensitive to the Al distribution, and in general, Al pairs in 8R windows lower the free-energy barrier for diffusion and stabilize the product configuration with two [Cu(NH_3_)_2_]^+^ cations in the same cage. This might be taken to suggest that the rate of the SCR-NOx reaction could be accelerated by selectively positioning the Al atoms as Al pairs. However, even though those results are well beyond the limits of traditional AIMD, the simulation cells employed are overly simple and lack realistic Al and NH_4_^+^ concentrations.

Unbiased MD simulations using time scales of multiple nanoseconds and supercells with over 2300 framework atoms at a variety of Si/Al ratios and Cu^+^, NH_4_^+^, and NH_3_ loadings show that [Cu(NH_3_)_2_]^+^ cations can visit on average 3 to 4 cages and diffuse as far as 30 Å in a few nanoseconds. They also show that long-range migration to remote cages requires the simultaneous displacement of a charge-compensating NH_4_^+^ cation. An excess of NH_3_ facilitates the movement of the positive charges via proton transfer from NH_4_^+^ to NH_3_, thus enhancing the long-range diffusion of [Cu(NH_3_)_2_]^+^ complexes.

Regarding catalytic activity, we observed that the probability of finding two [Cu(NH_3_)_2_]^+^ complexes in the same cage, which is necessary for the SCR-NOx reaction at low temperature, correlates directly with the Cu content and the Al content but not so much with the Al distribution. These trends were confirmed experimentally through testing the SCR-NOx reaction at low temperatures using Cu-CHA zeolites with different Si/Al and Cu/Al molar ratios, where we found increasing catalytic performance with increasing Al and Cu loading.

These results demonstrate the power of combining high-throughput DFT calculations, machine learning, and molecular dynamics simulations for simulating transport in nanoporous catalysts. The collection of the training data and the training of the NNP had a lower total computational cost than a single traditional AIMD simulation and resulted in scalable, fast, and accurate simulations.

This overall strategy is broadly applicable to other unsolved questions in nanoporous catalysts since it enables DFT accuracy and nanosecond-long MD simulations for thousands of atoms and possibly beyond by combining ML and enhanced sampling techniques.^82^

## Data Availability

The experimental and computational data that support the findings of this study are available from the corresponding author upon reasonable request. The data sets generated during this study are available at https://figshare.com/projects/Dataset_and_machine_learning_potential_Cu-CHA/167645. The code used for this study can be downloaded from https://github.com/learningmatter-mit/NeuralForceField.

## Supporting Information Available

Models, methods, and experimental section; Tables S1 to S4 with calculated energies, chemical compositions, and characterization of the experimental samples; Figures S1 to S8 showing the models, the collective variable, diffusion geometries, and trajectories; PXRD and FESEM characterization (PDF)
