# Supplementary material for: Effect of Framework Composition and NH3 on the Diffusion of Cu+ in Cu-CHA Catalysts Predicted by Machine-Learning Accelerated Molecular Dynamics
Source: ACS Cent Sci. 2023 Oct 18;9(11):2044–56. doi: 10.1021/acscentsci.3c00870 (PMC10683499; doi:10.1021/acscentsci.3c00870)
Supplement: Supplementary file 1 — oc3c00870_si_001.pdf [file oc3c00870_si_001.pdf]

Supporting Information

Effect of framework composition and NH_3 on the diffusion of Cu^+ in Cu-CHA catalysts predicted by machine-learning accelerated molecular dynamics

Reisel Millan,[†,‡] Estefanía Bello-Jurado,[¶] Manuel Moliner,[¶] Mercedes Boronat,[*,¶] and Rafael Gomez-Bombarelli[*,†]

†*Department of Materials Science and Engineering, Massachusetts Institute of Technology, Cambridge, MA 02139*

‡*Universitat Politècnica de València*

¶*Instituto de Tecnología Química, Universitat Politècnica de València-Consejo Superior de Investigaciones Científicas, Avenida de los Naranjos s/n, 46022 Valencia, Spain*

E-mail: boronat@itq.upv.es; rafagb@mit.edu

Contents

Methods **S2**

Catalyst Models S2

Zeolite synthesis S7

Characterization S9

Catalytic evaluation S9

References **S18**

Methods

Catalyst Models

The Cu-CHA catalytic system was modeled using three different supercells of increasing size (see Figure S1). The smallest system is a 1x1x1 hexagonal supercell of CHA framework containing 2Al, 34 Si and 72 O atoms, with lattice parameters, a = b = 13.81 Å, c = 15.00 Å, α = 89.86°, β = 89.94°, γ = 120.41°. Three models with different lattice distribution of the two Al atoms were created, and the two negative charges introduced by the presence of Al in the framework were compensated with two $[Cu(NH_3)_2]^+$ complexes (Figure 2a). These models were used to perform DFT-based umbrella sampling (US) molecular dynamics (MD) simulations and in the first generation of the NNP.

A 2x2x2 triclinic supercell containing 96 T and 192 O atoms, with lattice parameters a = 18.68 Å, b = 18.67 Å, c = 18.67 Å, α = 94.68°, β = 94.63°, γ = 94.72° was used for the subsequent training of the NN potentials including additional Al distributions, Al contents, and compensating species ($[Cu(NH_3)_2]^+$, $NH_4{}^+$ and H^+) as well as additional NH_3 molecules as summarized in Table 1. For the systems with 3 and 7 Al atoms, corresponding to Si/Al $\sim$ 31 and 13, respectively, ten random Al distributions were generated. For the systems with two Al atoms, corresponding to Si/Al = 47, ten different Al distributions were generated,

one with 2 Al in the same 4R, one with two Al in the same 6R, four of them containing 2 Al in the same 8R, and four of them with the 2 Al in different rings. Despite the Si/Al ratio in these models is clearly higher than in the industrial catalysts, they are included to represent accurately a diversity of specific local environments, in particular different Al pairs in the 8MR being crossed. As the number of Al atoms in the unit cell increases, the number of possible Al distributions increases too, making it difficult to have a certain control on this variable. Single-point DFT calculations and NNP US-MD simulations were performed using this model.

Finally, a 4x4x4 triclinic supercell containing 768 T and 1536 O atoms, with lattice parameters a = 37.35 Å, b = 37.38 Å, c = 37.34 Å, α = 94.64°, β = 94.59°, γ = 94.47 was employed to run NNP unbiased MD simulations on more realistic systems and reaction conditions. Three lattice compositions corresponding to Si/Al $\sim$ 30 (26 Al, 742 Si and 1536 O atoms), Si/Al $\sim$ 14 (50 Al, 718 Si and 1536 O atoms) and Si/Al $\sim$ 10 (50 Al, 718 Si and 1536 O atoms) were considered, and for each of them three random Al distributions were generated. For the model with the highest Al content (Si/Al$\sim$ 10), three additional Al distributions were generated in which all Al atoms were either forming pairs in the 6R units, forming pairs in the 8R windows, or heterogeneously distributed along the system so that Al-rich and Al-poor regions are found in the same unit cell.

The negative charges in each model were compensated with $[Cu(NH_3)_2]^+$, $NH_4{}^+$ and H^+ as summarized in Table S2. In the models with low Al content (L, Si/Al $\sim$ 30 and medium Al content (M, Si/Al $\sim$ 14), two Cu loadings were considered, 4 and 20 atoms per unit cell, corresponding to Cu spatial densities of 0.08 and 0.4 Cu/1000 Å^2 respectively and 0.5% and 2.6% Cu/Si ratios, of the order of experimental catalysts. The models are labeled with a letter indicating the Al content (L, M or H) followed by two numerical values indicating the number of $[Cu(NH_3)_2]^+$, and $NH_4{}^+$ compensating cations. Thus, L(20-6) corresponds to a low Al content (Si/Al $\sim$ 30) with 26 Al atoms in the unit cell compensated with 20 $[Cu(NH_3)_2]^+$ and 6 $NH_4{}^+$ cations. M(20-30) corresponds to a medium Al content (Si/Al

$\sim$ 14) with 50 Al in the unit cell compensated with 20 $[Cu(NH_3)_2]^+$ and 30 NH_4^+ cations. M(20-30H+) corresponds to a medium Al content (Si/Al $\sim$ 14) with 50 Al in the unit cell compensated with 20 $[Cu(NH_3)_2]^+$ and 30 H^+.

The models with high Al content (H, Si/Al $\sim$ 10) have the same composition H(20-48), that is, 68 Al in the unit cell compensated with 20 $[Cu(NH_3)_2]^+$ and 48 NH_4^+ cations. The labels in this case indicate four different Al distributions, with Al pairs in the same six-membered ring H(20-48)6R, with Al pairs in the same eight-membered ring H(20-48)8R, a random distribution of Al H(20-48)rand, and a biased, spatially-heterogeneous distribution containing regions with high and low Al content H(20-48)bias.

All DFT calculations (single points evaluations as well as the molecular dynamics simulations) were carried out at the revPBE+D3[1,2] level of theory with the software CP2K.[3] The Gaussian and Plane Waves (GPW) method[4] was used with the TZVP basis set for all atoms except Cu, which was described with the DZVP-MOLOPT-SR-GTH basis set. A cutoff energy of 400 Ry was used for the auxiliary plane waves and the core electrons were represented with GTH pseudopotentials.[5]

All NNP-MD simulations were performed using NNPs trained with the PaiNN[6] architecture. Regular NNP-MD simulations were run in the NVT ensemble at 500 K, and consisted of a production run of at least 3 ns after 100 ps of equilibration. The temperature was controlled by a Nosé–Hoover chain thermostat[7,8] with three beads and a time constant of 200 fs. The time step to integrate the equations of motion was set to 0.5 fs.

The mean squared displacements (MSD) obtained for $[Cu(NH_3)_2]^+$ and NH_4^+ were calculated via the following equation:

$$MSD(t) = \frac{1}{t_s - t + 1} \sum_{t_0=0}^{t_s=t} \frac{1}{N} \sum_{i=1}^{N} \left[r_i(t_0 + t) - r_i(t_0)\right]^2 \tag{1}$$

where N corresponds to the number of $[Cu(NH_3)_2]^+$ or NH_4^+ cations, t_s is the number of time steps in the simulation, the t_0 are the different time origins.

Helmholtz free energy profiles for the diffusion of $[Cu(NH_3)_2]^+$ species through the 8R windows of Cu-SSZ-13 zeolite were obtained from umbrella sampling (US) simulations[9] at 423 K. The success of the US technique relies on the choice of a collective variable (CV) that represents unambiguously the process considered. It has been shown in the literature that the collective variable (ξ) that best describes the diffusion of $[Cu(NH_3)_2]^+$ from one cavity A ($\xi < 0$) to another one B ($\xi > 0$) is the projection of the position vector of the Cu atom on the vector normal to the average plane of the 8R (see Figure S2). This collective variable takes the value $\xi = 0$ when the Cu atom is located in the plane of the 8R. In the biased simulations, the collective variable ξ was split into 40 (for DFT-based simulations) or 80 (for NN-based simulations) equidistant windows in the range ξ = -4 to ξ = 4. In each window, an independent biased MD simulation of 20 ps was run restricting the collective variable to one specific value but ensuring complete sampling of the configuration space in all other degrees of freedom. A harmonic bias potential centered at the corresponding value of the collective variable was applied to restrict the sampling to each window individually and to ensure sufficient overlap between the sampling of adjacent windows. The force constant of the harmonic potential was set to 12 kcal/mol/Å^2. All DFT-based biased simulations were performed with PLUMED[10] interfaced to the CP2K engine.

Neural network potential training

All NN potentials trained were based on the PaiNN architecture[6] which uses equivariant message-passing for the ground truth prediction. For the training we used three convolutions, a cutoff radius of 5Å, 32 Gaussian functions for distance expansion and 128-size vector for the atomic features. Neural network parameters were optimized using Adam algorithm with a batch size of 32, initial learning rate of 1×10^{-4}, learning decay of 0.5 and learning patience of 25.

The reference energies of Si, Al, Cu, N, O, and H were evaluated using the revPBE+D3 energies of the lowest energy structure of each composition. These energies were used to fit

a linear regression model with atomic contribution to the reference energy of each element as predictor variables. The reference energy was subtracted from the revPBE+D3 energy of every geometry in the training, test and validation sets. The forces were used without modifications. The training coefficients for the energies and the forces were set to 0.01 and 1.0, respectively.

The acquisition of training data was performed using active learning (AL) with a query-by-committee approach[11–18] as illustrated in Figure 1a. In this approach a committee (ensemble) of NNPs is trained on the available labelled data and new data is collected based on the maximum disagreement (variance) of the prediction the committee members.

The first generation of the potential was trained on a randomly collected subset of the DFT data generated from a previous study[19] and from three biased simulations performed with DFT at 423 K, used as reference ground truth. In total, there were ∼9000 geometries in the initial dataset. This pretrained potential was then retrained in 4 active learning loops using the 2x2x2 triclinic supercell described in previous section (see Figure S1. For each loop, biased MD trajectories were generated with the learned interatomic potential of the previous loop. The following temperatures were used in the active learning loops 298 K, 423 K, 500 K and 550 K. The bias was applied using a harmonic potential on the collective variable used in US. Each MD was run for 100 ps and geometries were collected every 100 fs. The selection of the new geometries from the MD trajectories was carried out using as criterion the force uncertainty from an ensemble of three NN potentials. The variances of the forces from the ensemble of potentials were ranked in descending order and the first geometries were selected to increase the dataset in ∼10% . The nonphysical geometries and those with low uncertainty, $<$ 2 kcal/mol were discarded. Up to this point, the dataset contained structures where the Al substitutions where compensated with Cu^+ as diaminecopper(I) so that the trained potential did not properly describe local environments of Al compensated with ${NH_4}^+$ or H^+. The acquisition of new geometries with new compositions including ${NH_4}^+$ and H^+ was performed using adversarial attack[20] for 6 more generations with NNP trained on the

last generation of AL. The initialization of adversarial attacks were performed by displacing each coordinate σ ~N(0,0.01 Å) of one optimized geometry for every chemical composition containing NH_4^+ and H^+. The resulting attack σ was optimized for 100 iterations using the Adam optimizer with learning rate of 0.0001 and with the normalized temperature kT set to 20 kcal/mol.

Then, five more generations of active learning was performed, with biased MD simulations at temperatures ranging from 600 to 1000 K. All models were trained by randomly partitioning the available data into training (60%), validation (20%) and test sets (20%). This splitting was perform for each composition and then the training, test and validation sets of each composition were combined in the final training, test and validation sets. Thus, the final training, test and validation sets had a balanced distribution of all chemical compositions.

Zeolite synthesis

CHA zeolite with a Si/Al~7 (CHA07)

5.91 g of a 25 wt% aqueous solution of N,N,N-trimethyl-1-adamantylammoniumhydroxide (TMAda, Sachem) was added to 5.41 g of deionized H_2O. Next, 0.11 g of $Al(OH)_3$ (76.5 wt%, Thermo Fisher) and 0.85 g of a 5M sodium hydroxide solution (NaOH: 16.7 wt% NaOH in deionized water; NaOH pellets 98 wt%, Alfa Aesar) was added to the aqueous TMAdaOH solution and the mixture was stirred under ambient conditions for 15 minutes. Finally, 2.13 g of colloidal silica (Ludox HS40, 40 wt%, Sigma Aldrich) was added to the mixture and stirred for 2 h under ambient conditions. The final gel composition was: SiO_2 : 0.056 Al_2O_3 : 0.49 TMAdaOH : 0.25 NaOH : 46.5 H_2O. The resultant gel was charged into a stainless steel autoclave with a Teflon liner. The crystallization was then conducted at 160 °C for 6 days under dynamic conditions. The solid product was filtered, washed with abundant water, and dried at 100 °C. The solids were calcined at 580 °C for 5 h in air.

CHA zeolite with a Si/Al∼13 (CHA13)

5.89 g of a 25 wt% aqueous solution of N,N,N-trimethyl-1-adamantylammoniumhydroxide (TMAda, Sachem) was added to 5.42 g of deionized H_2O. Next, 0.042 g of $Al(OH)_3$ (76.5 wt%, Thermo Fisher) and 0.86 g of a 5M sodium hydroxide solution (NaOH: 16.7 wt% NaOH in deionized water; NaOH pellets 98 wt%, Alfa Aesar) was added to the aqueous TMAdaOH solution and the mixture was stirred under ambient conditions for 15 minutes. Finally, 2.10 g of colloidal silica (Ludox HS40, 40 wt%, Sigma Aldrich) was added to the mixture and stirred for 2 h under ambient conditions. The final gel composition was: SiO_2 : 0.02 Al_2O_3 : 0.50 TMAdaOH : 0.26 NaOH : 47H_2O. The resultant gel was charged into a stainless steel autoclave with a Teflon liner. The crystallization was then conducted at 160 °C for 6 days under dynamic conditions. The solid product was filtered, washed with abundant water, and dried at 100 °C. The solids were calcined at 580 °C for 5 h in air.

CHA zeolite with a Si/Al∼23 (CHA23)

1.55 g of FAU zeolite (FAU, CBV760 with Si/Al=26, Zeolyst, lot number: 76004N002648) was added to 6.74 g of a 25 wt% aqueous solution of N,N,N-trimethyl-1-adamantylammoniumhydroxide (TMAda, Sachem). The mixture was maintained under stirring the required time to evaporate the excess of water until achieving the desired gel concentration. The final gel composition was: SiO_2 : 0.019 Al_2O_3 : 0.4 TMadaOH : 5 H_2O. The resultant gel was charged into a stainless steel autoclave with a Teflon liner. The crystallization was then conducted at 175 °C for 12 days under static conditions. The solid product was filtered, washed with abundant water, and dried at 100 °C. The solids were calcined at 580 °C for 5 h in air.

Cu-exchange treatments

The Na-containing CHA calcined solids were first exchanged with a 2M aqueous solution of ammonium nitrate (NH_4Cl, Sigma-Aldrich, 99% by weight) with a liquid/solid ratio of 10, maintaining the mixture at 80 °C for 2 hours under agitation. Afterwards, the solids were

recovered by filtration. 0.3 g of the Na-free zeolites was introduced in 30 ml of an aqueous solution of $Cu(CH_3COO)_2{\cdot}H_2O$ [14.1 mg of $Cu(CH_3COO)_2{\cdot}H_2O$ dissolved in 30 ml of water or 28.3 mg of $Cu(CH_3COO)_2{\cdot}H_2O$ dissolved in 30 ml of water for materials containing ∼1.5 or 3%wt Cu, respectively], maintaining a solid/liquid ratio of 10 g/l at 80 °C for 24 h. Finally, the solids were filtered and washed with distilled water, dried and calcined at 550 °C in air for 4 h.

Characterization

Powder X-ray diffraction (PXRD) measurements were performed with a multi sample Philips X'Pert diffractometer equipped with a graphite monochromator, operating at 40 kV and 35 mA, and using Cu Kα radiation (λ = 0.1542 nm). The PXRD patterns reveal the good crystallization of the CHA materials (see Figure S7 in the Supporting Information), all of them presenting particle sizes within the sub-micron scale (below 1 µm, see Figure S7 and Table S3 in the Supporting Information). Chemical analyses were carried out in a Varian 715-ES ICP-Optical Emission spectrometer, after solid dissolution in HNO_3/HCl/HF aqueous solution. Nitrogen adsorption isotherms at -196 °C were measured on a Micromeritics ASAP 2020 with a manometric adsorption analyser to determinate the textural properties of the samples. The morphology of the samples was studied by field emission scanning electron microscopy (FESEM) using a ZEISS Ultra-55 microscope.

Catalytic evaluation

The catalytic activity was evaluated for the selective catalytic reduction (SCR) of NOx with NH_3 in a fixed bed, quartz tubular reactor with ∼1 cm inner diameter. 20 mg sieve fractionated catalysts (200-400 µm) were placed between 0.6 of silicon carbide fractions (SiC, Fisher Chemical, 200-400 µm) generating an overall bed height of ∼ 1.2 cm. The catalysts were introduced in the reactor and heated up to 550 °C in a 300 mL/min flow of nitrogen and maintained at this temperature for one hour. Afterwards, the feed was admitted over

the catalyst with an overall flow of 600 mL/min. The feed composition for the catalytic tests performed over the Cu-containing catalysts was 500 ppm NO, 550 ppm NH3, 7% O2 and 5% H2O, resulting in a very high space velocity (GHSV=1800000 ml/h.grcat). The evaluated reaction temperatures were 190, 180 and 170 °C. The conversion of NO was measured under steady state conversion at each temperature using a chemiluminescence detector (Thermo 62C). The TOF were estimated by dividing the moles of NO molecules converted per second by the moles of Cu atoms in the catalysts, according to

$$TOF = \frac{moles\ NO\ converted/s}{moles\ Cu} \tag{2}$$

From the low temperature NO conversion results, the rate constants (k) can be calculated using a first-order kinetic equation, as described previously in the literature:[21]

$$k = -\frac{F_0}{[NO]_0 W} \ln(1 - X) \tag{3}$$

where F_0 is the molar NO feed rate, $[NO]_0$ is the molar concentration at the inlet, W is the catalyst amount (gr) and X is the NO conversion. The Arrhenius equation was employed to estimate the apparent activation energies (Ea) after its linearization as follows:

$$k = Ae^{-E_a/RT} \tag{4}$$

$$\ln k = \ln A - \frac{E_a}{R}\left(\frac{1}{T}\right) \tag{5}$$

where A is the pre-exponential factor, R is the universal gas constant and T denotes the absolute temperature associated with the reaction (in Kelvin).

No unexpected or unusually high safety hazards were encountered.

Table S1: Free energies of activation (ΔF_{act}) and reaction (ΔF) for the diffusion of a $[Cu(NH_3)_2]^+$ complex between two neighboring cages through 8R windows with different Al distribution obtained from NNP-based biased simulations at 423 K.

Al distribution	ΔF_{act} (kcal/mol)	ΔF (kcal/mol)
SR1	3.9	0.4
SR2	4.6	1.7
SR3	5.4	2.3
SR4	4.1	0.5
DR1	6.1	3.2
DR2	6.7	2.9
DR3	6.4	3.2
DR4	5.4	-0.7
S4R	7.3	4.5
S6R	6.4	5.4

Table S2: Chemical composition, cationic species and molecules included in the triclinic $T_{768}O_{1536}$ supercell models used for NNP-based regular molecular dynamics simulations.

Name	Formulas	Si/Al	Al	Si	$[Cu(NH_3)_2]^+$	NH_4^+	NH_3	H^+
L(20-6)	$H_{144}Al_{26}Cu_{20}N_{46}O_{1536}Si_{742}$	28.5	26	742	20	6	0	0
L(4-22)	$H_{112}Al_{26}Cu_{4}N_{30}O_{1536}Si_{742}$	28.5	26	742	4	22	0	0
M(20-30)	$H_{240}Al_{50}Cu_{20}N_{70}O_{1536}Si_{718}$	14.3	50	718	20	30	0	0
M(4-46)	$H_{208}Al_{50}Cu_{4}N_{54}O_{1536}Si_{718}$	14.3	50	718	4	46	0	0
M(20-30H+)	$H_{150}Al_{50}Cu_{20}N_{40}O_{1536}Si_{718}$	14.3	50	718	20	0	0	30
M(20-30)-NH3	$H_{423}Al_{50}Cu_{20}N_{131}O_{1536}Si_{718}$	14.3	50	718	20	30	60	0
$H(20\text{-}48)_{6R}$	$H_{312}Al_{68}Cu_{20}N_{88}O_{1536}Si_{700}$	10.3	68	700	20	48	0	0
$H(20\text{-}48)_{8R}$	$H_{312}Al_{68}Cu_{20}N_{88}O_{1536}Si_{700}$	10.3	68	700	20	48	0	0
$H(20\text{-}48)_{\text{rand}}$	$H_{312}Al_{68}Cu_{20}N_{88}O_{1536}Si_{700}$	10.3	68	700	20	48	0	0
$H(20\text{-}48)_{\text{bias}}$	$H_{312}Al_{68}Cu_{20}N_{88}O_{1536}Si_{700}$	10.3	68	700	20	48	0	0

Table S3: Chemical composition and physico-chemical properties of the synthesized CHA zeolites.

Sample	Si/Al	Al/*cha* cage	Crystal size (nm)	BET surface area (m^2/g)	Micropore area (m^2/g)	Micropore volume (cm^3/g)
CHA07	7.3	1.4	~100	562	532	0.26
CHA13	12.7	0.9	~400-800	556	551	0.27
CHA23	23.3	0.5	~100	550	490	0.25

Table S4: Chemical composition of the Cu-exchanged CHA zeolites.

**Sample**	**Si/Al**	**%wt Cu**	**Cu/Al**	**Al/*cha* cage**
CHA07_1.5Cu	7.3	1.58	0.11	0.16
CHA07_3.0Cu	7.3	2.92	0.20	0.30
CHA13_1.5Cu	12.7	1.55	0.18	0.17
CHA23_1.5Cu	23.3	1.54	0.35	0.17

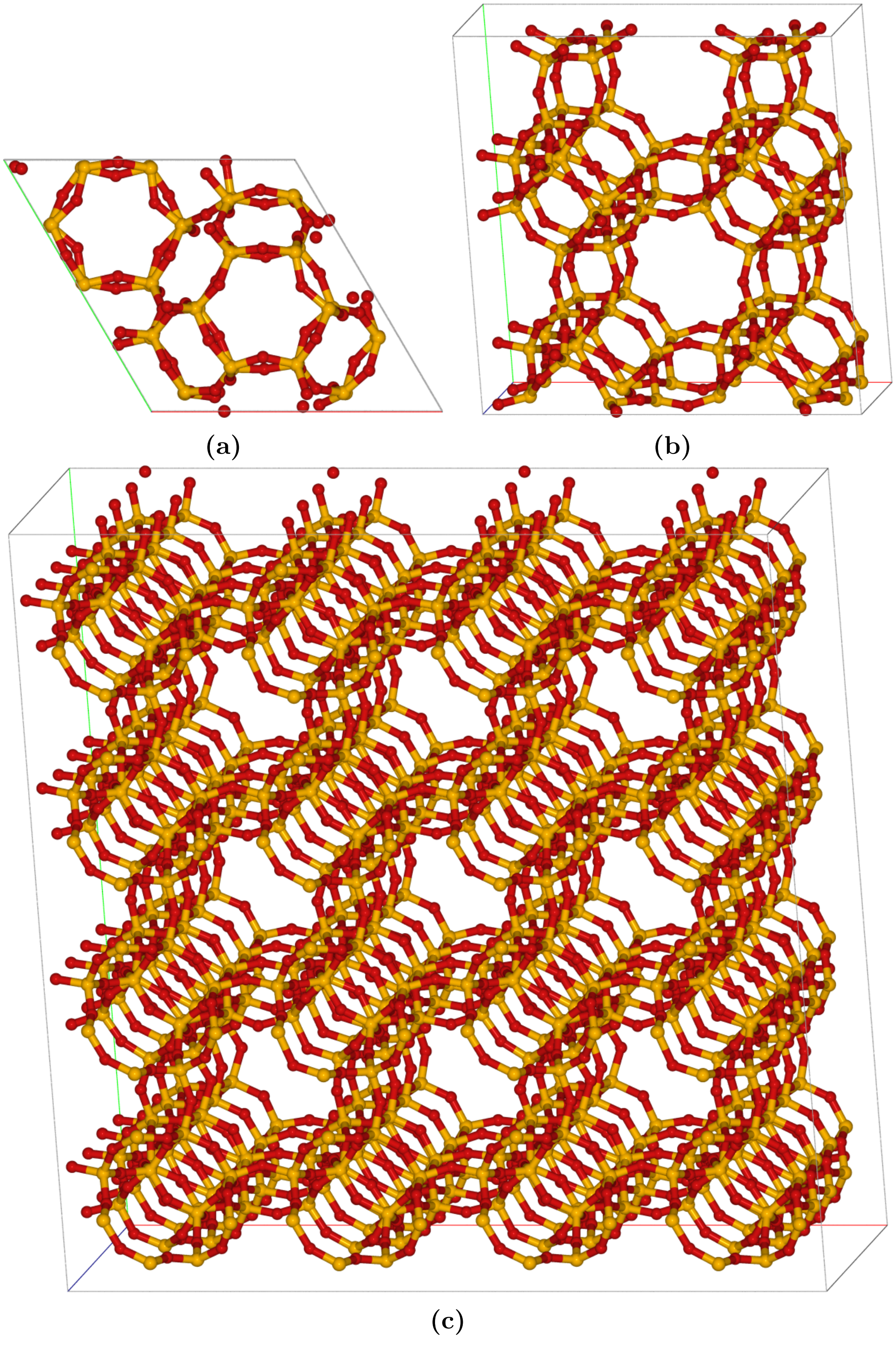

Figure S1: Representation of the three types of base lattices used for umbrella sampling simulations (a and b) and for unbiased MD simulations (c). The representations correspond the all-silica CHA, however, the models used in this work include different combinations of Al number and locations starting from these base lattices, as explained in the main text. Color code: Si and O are depicted as orange and red balls, respectively.

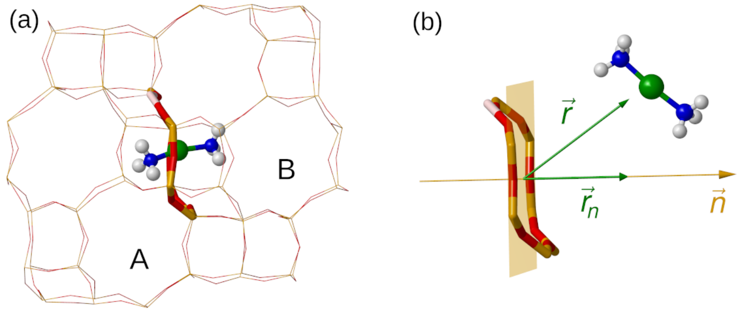

Figure S2: (a)Diffusion of $[Cu(NH_3)_2]^+$ complex from cage A ($\xi < 0$) to cage B ($\xi > 0$). (b) Representation of the collective variable ξ describing the $[Cu(NH_3)_2]^+$ diffusion through the 8R window (transparent yellow plane)

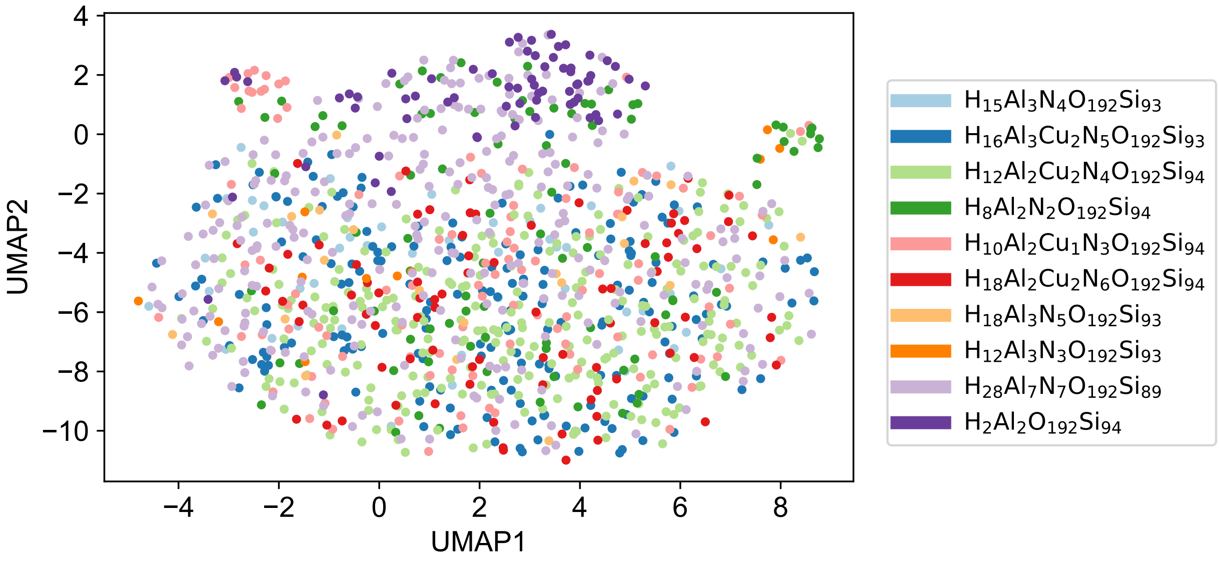

Figure S3: UMAP plot for the feature vectors of Al atoms from ∼1% of the dataset. Both axes are on the same scale.

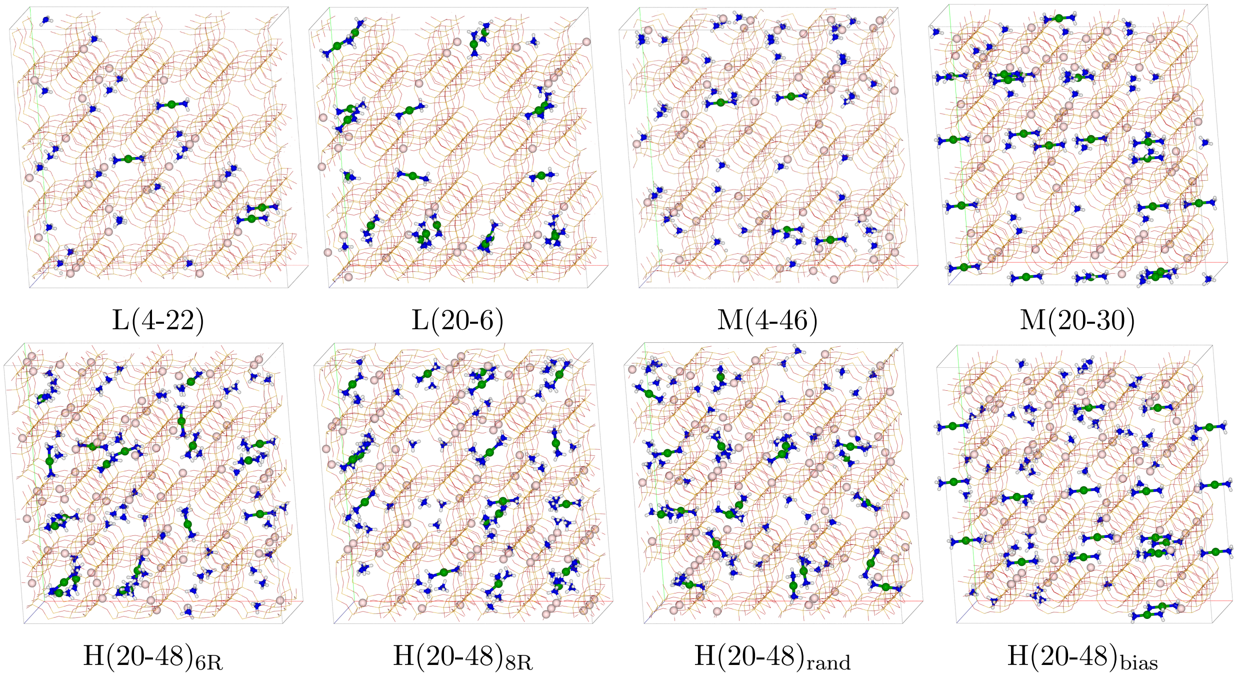

Figure S4: Snapshots of the input structures corresponding to the eight different zeolite framework compositions considered in the NNP unbiased MD simulations. The first letter indicates the Al content, low (L, Si/Al $\sim$ 30) with 26 Al in the unit cell, medium (M, Si/Al $\sim$ 14) with 50 Al in the unit cell, and high (H, Si/Al $\sim$ 10). Then, the following two numerical values indicate the number of $[Cu(NH_3)_2]^+$, and $NH_4{}^+$ compensating cations. The subscripts in the bottom models that contain 20 Cu atoms per unit cell indicates the Al distribution, forming pairs in 6R or 8R, random (rand) and biased. Si, O, Al, H, Cu and N atoms are depicted as orange, red, light brown, white, green and blue.

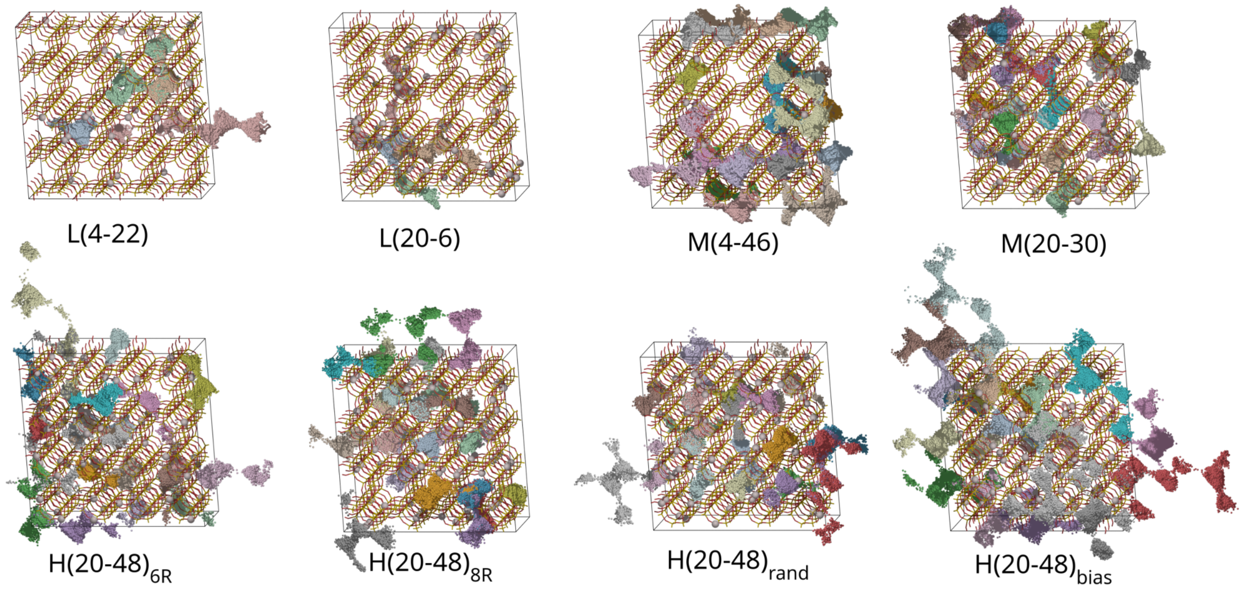

Figure S5: Scatter plots showing the regions visited by Cu^+ in the simulations run at 500 K for 5 ns. Color code: Si, O, and Al atoms are depicted as orange, red and light brown. Each Cu^+ cation is represented with a random color.

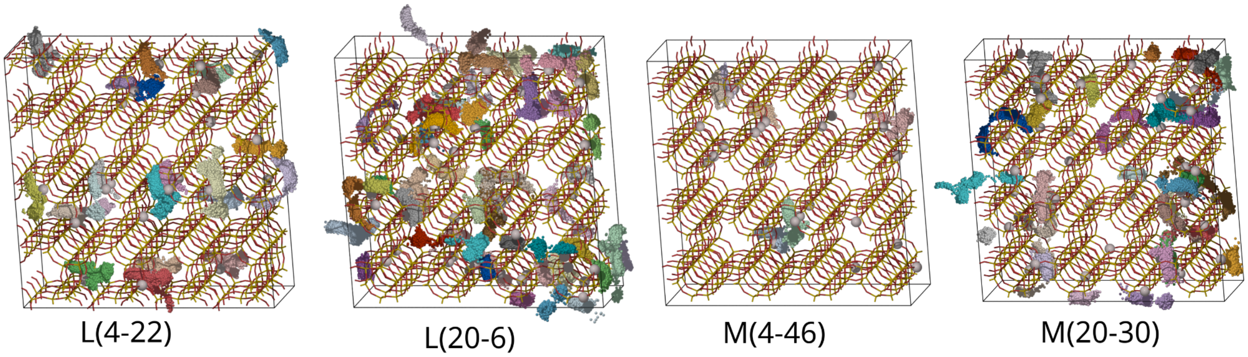

Figure S6: Scatter plots showing the regions visited by NH_4^+ in the simulations run at 500 K for 5 ns. Color code: Si, O, and Al atoms are depicted as orange, red and light brown. Each NH_4^+ is represented with a random color.

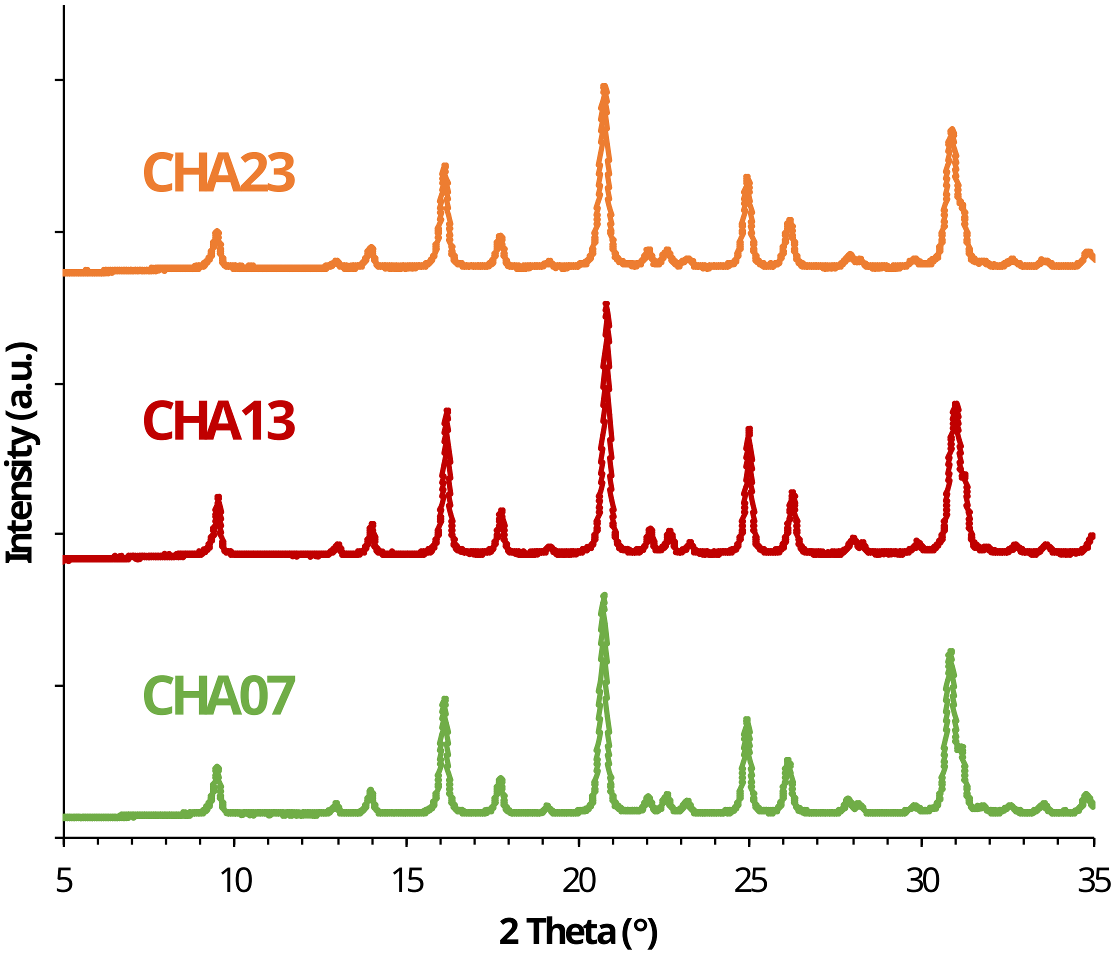

Figure S7: PXRD patters of the synthesized CHA zeolites

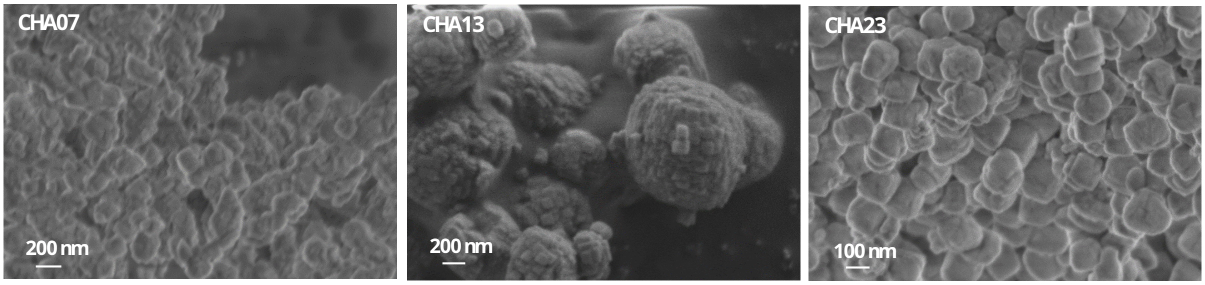

Figure S8: FESEM images of the synthesized CHA zeolites

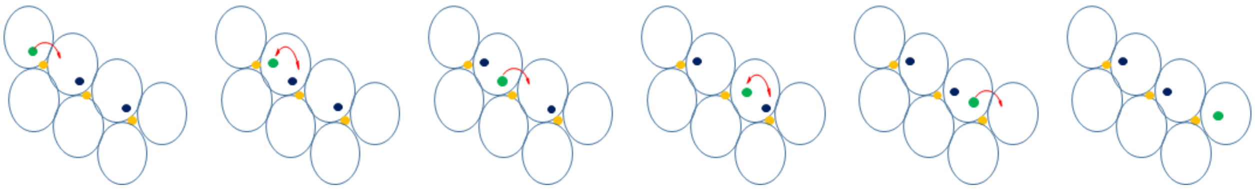

Scheme S1: Representation of long-range $[Cu(NH_3)_2]^+$ migration assisted by NH_4^+. Al, Cu^+ and NH_4^+ are represented with yellow, green and blue balls, respectively.

References

(1) Zhang, Y.; Yang, W. Comment on "Generalized Gradient Approximation Made Simple". *Physical Review Letters* **1998**, *80*, 890.

(2) Grimme, S.; Antony, J.; Ehrlich, S.; Krieg, H. A consistent and accurate ab initio parametrization of density functional dispersion correction (DFT-D) for the 94 elements H-Pu. *The Journal of Chemical Physics* **2010**, *132*, 154104.

(3) Vandevondele, J.; Krack, M.; Mohamed, F.; Parrinello, M.; Chassaing, T.; Hutter, J. Quickstep: Fast and accurate density functional calculations using a mixed Gaussian and plane waves approach. *Computer Physics Communications* **2005**, *167*, 103–128.

(4) Lippert, G.; Hutter, J.; Parrinello, M. The Gaussian and augmented-plane-wave density functional method for ab initio molecular dynamics simulations. *Theoretical Chemistry Accounts 1999 103:2* **1999**, *103*, 124–140.

(5) Goedecker, S.; Teter, M. Separable dual-space Gaussian pseudopotentials. *Physical Review B* **1996**, *54*, 1703.

(6) Schütt, K. T.; Unke, O. T.; Gastegger, M. Equivariant message passing for the prediction of tensorial properties and molecular spectra. 2021; preprint, `https://arxiv.org/abs/2102.03150`.

(7) Martyna, G. J.; Klein, M. L.; Tuckerman, M. Nosé-Hoover chains: The canonical ensemble via continuous dynamics. *The Journal of Chemical Physics* **1992**, *97*, 2635–2643.

(8) Hoover, W. G.; Ladd, A. J. C.; Moran, B. High-Strain-Rate Plastic Flow Studied via Nonequilibrium Molecular Dynamics. *Physical Review Letters* **1982**, *48*, 1818–1820.

(9) Torrie, G. M.; Valleau, J. P. Nonphysical sampling distributions in Monte Carlo free-energy estimation: Umbrella sampling. *Journal of Computational Physics* **1977**, *23*, 187–199.

(10) Tribello, G. A.; Bonomi, M.; Branduardi, D.; Camilloni, C.; Bussi, G. PLUMED 2: New feathers for an old bird. *Computer Physics Communications* **2014**, *185*, 604–613.

(11) Behler, J. Constructing high-dimensional neural network potentials: A tutorial review. *International Journal of Quantum Chemistry* **2015**, *115*, 1032–1050.

(12) Smith, J. S.; Nebgen, B.; Lubbers, N.; Isayev, O.; Roitberg, A. E. Less is more: Sampling chemical space with active learning. *Journal of Chemical Physics* **2018**, *148*, 241733.

(13) Schran, C.; Brezina, K.; Marsalek, O. Committee neural network potentials control generalization errors and enable active learning. *Journal of Chemical Physics* **2020**, *153*, 104105.

(14) Musil, F.; Willatt, M. J.; Langovoy, M. A.; Ceriotti, M. Fast and Accurate Uncertainty Estimation in Chemical Machine Learning. *Journal of Chemical Theory and Computation* **2019**, *15*, 906–915.

(15) Peterson, A. A.; Christensen, R.; Khorshidi, A. Addressing uncertainty in atomistic machine learning. *Physical Chemistry Chemical Physics* **2017**, *19*, 10978–10985.

(16) Lookman, T.; Balachandran, P. V.; Xue, D.; Yuan, R. Active learning in materials

science with emphasis on adaptive sampling using uncertainties for targeted design. *npj Computational Materials* **2019**, *5*, 21.

(17) Shapeev, A.; Gubaev, K.; Tsymbalov, E.; Podryabinkin, E. In *Machine Learning Meets Quantum Physics*; Schütt, K. T., Chmiela, S., von Lilienfeld, O. A., Tkatchenko, A., Tsuda, K., Müller, K.-R., Eds.; Springer International Publishing: Cham, 2020; pp 309–329.

(18) Imbalzano, G.; Zhuang, Y.; Kapil, V.; Rossi, K.; Engel, E. A.; Grasselli, F.; Ceriotti, M. Uncertainty estimation for molecular dynamics and sampling. *The Journal of Chemical Physics* **2021**, *154*, 74102.

(19) Millan, R.; Cnudde, P.; Speybroeck, V. v.; Boronat, M. Mobility and Reactivity of Cu+ Species in Cu-CHA Catalysts under NH3-SCR-NOx Reaction Conditions: Insights from AIMD Simulations. *JACS Au* **2021**, *1*, 1778–1787.

(20) Schwalbe-Koda, D.; Tan, A. R.; Gómez-Bombarelli, R. Differentiable sampling of molecular geometries with uncertainty-based adversarial attacks. *Nature Communications* **2021**, *12*, 1–12.

(21) Long, R. Q.; Yang, R. T. Selective Catalytic Reduction of NO with Ammonia over Fe3+-Exchanged Mordenite (Fe–MOR): Catalytic Performance, Characterization, and Mechanistic Study. *Journal of Catalysis* **2002**, *207*, 274–285.
